# Combined genetic-pharmacologic inactivation of tightly linked ADAMTS proteases in temporally specific windows uncovers distinct roles for versican proteolysis and glypican-6 in cardiac development

**DOI:** 10.1016/j.matbio.2024.05.003

**Published:** 2024-05-13

**Authors:** Timothy J. Mead, Sumit Bhutada, Simon J. Foulcer, Niccolò Peruzzi, Courtney M. Nelson, Deborah E. Seifert, Jonathan Larkin, Karin Tran-Lundmark, Jorge Filmus, Suneel S. Apte

**Affiliations:** a Department of Biomedical Engineering, Cleveland Clinic Lerner Research Institute, Cleveland, OH, USA; b Department of Pediatrics, Case Western Reserve University School of Medicine, Cleveland, OH, USA; c University Hospitals Rainbow Babies and Children’s Hospital, Cleveland, OH, USA; d Department of Experimental Medical Science, and Wallenberg Center for Molecular Medicine Lund University and The Pediatric Heart Center, Skane University Hospital, Lund, Sweden; e SynOA Therapeutics, Philadelphia, PA 19119, USA; f Sunnybrook Research Institute and Department of Medical Biophysics, University of Toronto, Toronto, Ontario, Canada

**Keywords:** Proteoglycan, Metalloprotease, Versican, Glypican, Terminomics, Degradomics

## Abstract

Extracellular matrix remodeling mechanisms are understudied in cardiac development and congenital heart defects. We show that matrix-degrading metalloproteases ADAMTS1 and ADAMTS5, are extensively co-expressed during mouse cardiac development. The mouse mutants of each gene have mild cardiac anomalies, however, their combined genetic inactivation to elicit cooperative roles is precluded by tight gene linkage. Therefore, we coupled *Adamts1* inactivation with pharmacologic ADAMTS5 blockade to uncover stage-specific cooperative roles and investigated their potential substrates in mouse cardiac development. ADAMTS5 blockade was achieved in *Adamts1* null mouse embryos using an activity-blocking monoclonal antibody during distinct developmental windows spanning myocardial compaction or cardiac septation and outflow tract rotation. Synchrotron imaging, RNA in situ hybridization, immunofluorescence microscopy and electron microscopy were used to determine the impact on cardiac development and compared to *Gpc6* and ADAMTS-cleavage resistant versican mutants. Mass spectrometry-based N-terminomics was used to seek relevant substrates. Combined inactivation of ADAMTS1 and ADAMTS5 prior to 12.5 days of gestation led to dramatic accumulation of versican-rich cardiac jelly and inhibited formation of compact and trabecular myocardium, which was also observed in mice with ADAMTS cleavage-resistant versican. Combined inactivation after 12.5 days impaired outflow tract development and ventricular septal closure, generating a tetralogy of Fallot-like defect. N-terminomics of combined ADAMTS knockout and control hearts identified a cleaved glypican-6 peptide only in the controls. ADAMTS1 and ADAMTS5 expression in cells was associated with specific glypican-6 cleavages. Paradoxically, combined ADAMTS1 and ADAMTS5 inactivation reduced cardiac glypican-6 and outflow tract *Gpc6* transcription. Notably, *Gpc6*^−/−^ hearts demonstrated similar rotational defects as combined ADAMTS inactivated hearts and both had reduced hedgehog signaling. Thus, versican proteolysis in cardiac jelly at the canonical Glu^441^-Ala^442^ site is cooperatively mediated by ADAMTS1 and ADAMTS5 and required for proper ventricular cardiomyogenesis, whereas, reduced glypican-6 after combined ADAMTS inactivation impairs hedgehog signaling, leading to outflow tract malrotation.

## Introduction

Congenital heart defects occur in over 1 % of livebirths worldwide (about 40,000 newborns annually in the United States) and result from diverse gene mutations [1–3]. Simple anomalies such as atrial or ventricular septal defect (VSD) and isolated valve stenosis as well as more complex malformations such as tetralogy of Fallot (ToF) can occur. ToF, a serious developmental anomaly occurring in 2 of every 10,000 live births, accounts for up to 10 % of congenital heart defects and comprises VSD, pulmonic trunk stenosis, rightward deviation of the aorta, leading to overriding aorta and right ventricular hypertrophy [4]. The majority of mutations causing cardiac developmental defects occur in genes encoding transcription factors, soluble signaling mediators and their receptors or cardiac contractile proteins [5]. Although the extracellular matrix (ECM) is a major component of the developing heart, relatively few ECM genes are implicated in cardiac development and the role of ECM, other than in valve development [6], has received relatively little attention.

The heart is formed as a linear vascular endothelium (endocardium)-lined tube that is converted to a four-chambered structure by looping and septation. Ventricular growth, compaction and trabeculation occur throughout cardiac development, but the process is particularly active from 9.5 to 12.5 days of gestation [7]. Subsequently, rotation of the outflow tract aligns the great vessels with their respective ventricular chambers [8]. Early cardiomyocytes and mesenchymal cells in cardiac cushions are surrounded by primordial ECM, termed cardiac jelly [7]. This highly hydrated ECM is enriched in the glycosaminoglycan hyaluronan (HA) complexed with large chondroitin-sulfate proteoglycans, primarily versican, with low levels of aggrecan also reported [7]. HA-proteoglycan complexes are stabilized by cartilage link protein, encoded by *Hapln1* [9]. Mouse mutants lacking versican or HA do not survive past 10 days of gestation because of arrested cardiac morphogenesis [10–12]. In addition to being a key part of the large proteoglycan complex during cardiac development, versican stabilizes HA and protects it from degradation [13,14]. The importance of the HA-versican complex in cardiac jelly is evident from the striking failure of myocardial growth and premature myocardial compaction when either versican or HA are lacking [13].

ADAMTS proteases, a family of 19 secreted metalloproteases [15], are major participants in versican remodeling and ADAMTS1 was previously implicated in ventricular cardiac jelly regression [7,16]. *Adamts1* and *Adamts5* mouse mutants each have known cardiac anomalies, specifically, impaired ventricular compaction in the former, and a pulmonic valve sculpting defect and aortic aggrecan accumulation in the latter [17–20]. ADAMTS1 and ADAMTS5 are phylogenetically closely related [21] and have overlapping proteolytic activity [22], suggesting potential cooperative roles in cardiac development. In this context, ADAMTS proteases including ADAMTS5, were previously shown to cooperate in versican proteolysis during mouse interdigit web regression [23] and ADAMTS9 and ADAMTS20 in palate closure [24] using combined mutants of the respective mouse genes.

To test a combined role for ADAMTS1 and ADAMTS5, we undertook pharmacological ADAMTS5 inactivation in *Adamts1* null embryos using a function-blocking antibody which was originally generated to target ADAMTS5 in osteoarthritis [25]. The findings of this hybrid-inactivation strategy, supported by new findings in a recently described cleavage-resistant *Vcan* mouse mutant [26] underscore the importance of versican proteolysis in cardiac jelly remodeling and myocardial development. We show that cooperation between ADAMTS1 and ADAMTS5 extends to regulation of glypican-6, discovered by application of N-terminomics for discovery of novel substrates. Taken together with new findings in *Gpc6* mutant hearts concordant with the effect of ADAMTS1 +ADAMTS5 combined inactivation, we propose a role for ADAMTS1 and ADAMTS5 acting via glypican-6, in promotion of hedgehog signaling during cardiac outflow tract rotation.

## Results

### *Adamts1* and *Adamts5* are co-expressed during cardiac development

Analysis of single cell RNA sequencing data (https://marionilab.cruk.cam.ac.uk/MouseGastrulation2018/) of mouse embryos from gastrulation to early organogenesis (8.5 days) [27], showed that at 7.75 and 8.0 days of gestation (E7.75 and E8), *Adamts1,* but not *Adamts5* was expressed in the earliest cardiomyocytes. At E8.25 and E8.5, *Adamts1* and *Adamts5* were each expressed in vascular endothelium and cardiomyocytes, with *Adamts1* expressed more widely at both stages [27]. We investigated *Adamts1* and *Adamts5* mRNA distribution in the developing heart from E10.5 – E16.5 by RNAscope in situ hybridization (Figure S1), with *Adamts1* expression additionally defined by β-galactosidase staining enabled by intragenic lacZ in *Adamts1* mutants (Figure S2). Both genes were expressed in the ventricular myocardium and endocardium from E10.5 – E12.5 (Figure S1). Whereas *Adamts1* was expressed in both the endocardium and endocardial cushion mesenchyme at E10.5, *Adamts5* was more robustly expressed by the cushion endocardium, especially their contact regions, and the cushion mesenchyme. At E12.5 and E14.5, *Adamts1* was expressed in the developing aortic valve cushion mesenchyme whereas *Adamts5* was expressed strongly in the cushion endocardial lining, with sporadic expressing cells seen in the cushion mesenchyme (Figure S1). At E16.5, *Adamts1* expression was stronger in aortic valve endocardium. Smooth muscle cells of the aorta showed strong expression of both genes from E12.5 onward, most evident in E16.5 aorta (Figure S1). Prior work had shown *Adamts5* expression in the aortic and pulmonic valve mesenchyme as well as in endocardium, myocardium and pericardium at E16.5 [17,28].

**Fig. 1.**
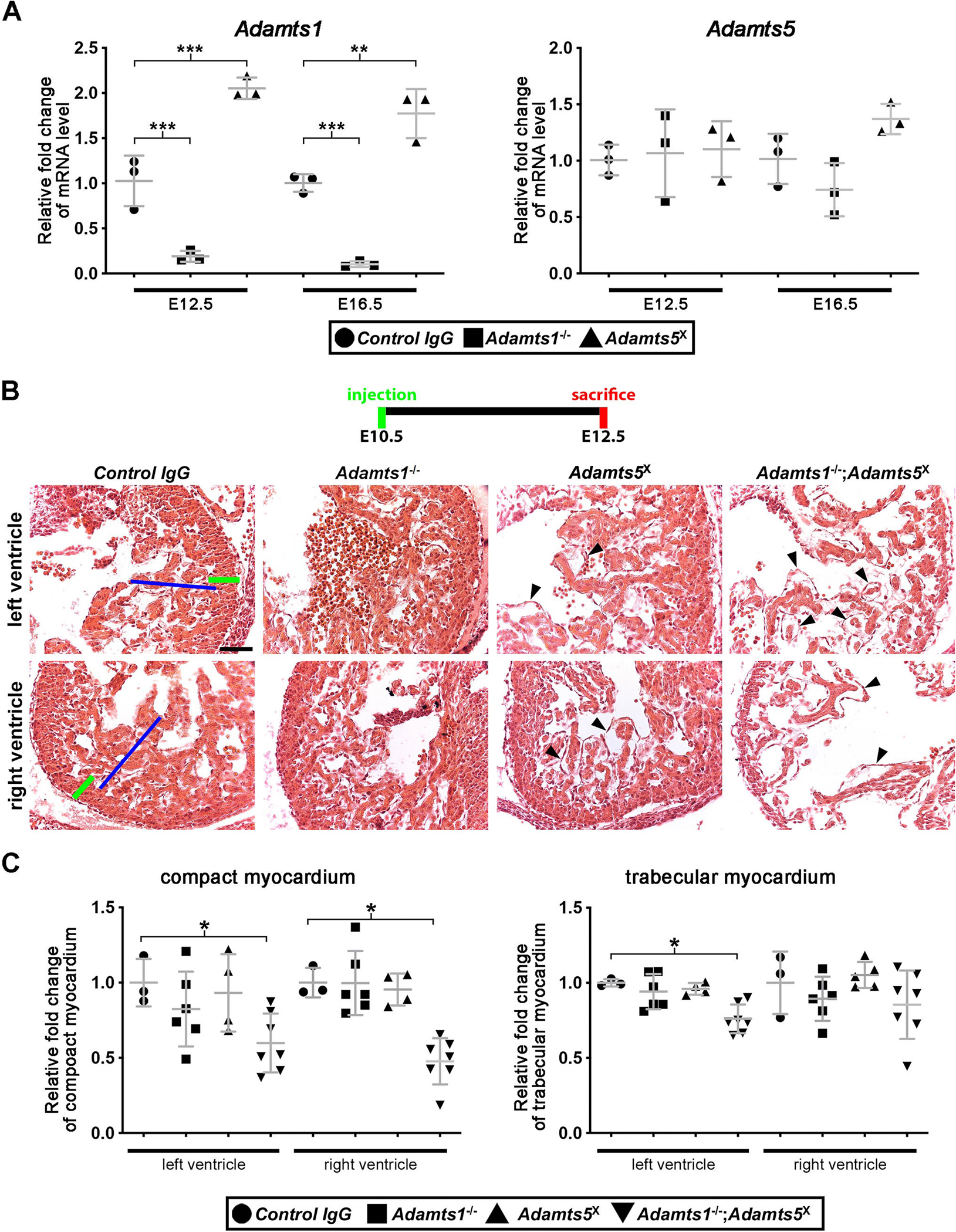
ADAMTS1 and ADAMTS5 cooperate in heart development. **A.** RT-qPCR analysis of *Adamts1* and *Adamts5* mRNA in control, *Adamts1*^−/−^; and *Adamts5*^X^ hearts showing elevated *Adamts1* mRNA in *Adamts5*^X^ hearts whereas *Adamts5* mRNA is unchanged in *Adamts1*^−/−^; hearts. *N* = 3 for each group. **B, C.** E12.5 hematoxylin-eosin-stained hearts (B) showed reduced thickness of compact (green line) and trabecular myocardium (blue line) in *Adamts1*^−/−^;*Adamts5*^X^ hearts, quantified in C. *N* = 3 control, 6 *Adamts1*^−/−^;, 4 *Adamts5*^X^, 7 *Adamts1*^−/−^;*Adamts5*^X^ hearts. Arrowheads in (B) depict swelling and apparent “bubbles” between trabecular myocardium and endocardium representing accumulated sub-endocardial cardiac jelly. Error bars represent ± SEM. *p ≤ 0.05; **p ≤ 0.01; ***p ≤ 0.001, Student *t*-test. Scale bar = 50μm.

The overlapping and proximate mRNA expression of this gene pair across the cardiac development timeline led us to test the possibility of transcriptional adaptation [29], i.e., compensatory upregulation of one gene after inactivation of the other, using qRT-PCR analysis of E16.5 mRNA from wild type and *Adamts1*^−/−^ whole hearts as well as hearts taken after targeting with the ADAMTS5 antibody. *Adamts1* mRNA was absent in *Adamts1*^−/−^ hearts and increased in ADAMTS5 antibody-inactivated hearts, which may potentially buffer the effects of individual knockout, since each protease has well-documented proteolytic activity against shared substrates such as aggrecan and versican (Fig. 1A) [30–33]. In contrast, *Adamts5* mRNA levels were unaffected by *Adamts1* inactivation.

Tight linkage of *Adamts1* and *Adamts5* loci (Figure S3A) precluded their combined inactivation by the traditional approach of interbreeding the respective mutants, i.e., their loci are only ~60 kb apart on mouse chromosome 16, predicting a low recombination probability [34,35]. Therefore, we combined genetic inactivation of *Adamts1* with pharmacologic inactivation of ADAMTS5. First, we tested the effectiveness of the well-characterized, high affinity function-blocking antibody GSK12F4.1H7 to effectively disrupt ADAMTS5 function during embryonic development by intraperitoneal (i.p.) injection of wild type pregnant dams at E12.5. Analysis of the hearts from embryos obtained at E16.5 demonstrated impaired pulmonic valve sculpting, previously described in *Adamts5*^−/−^ mice [17] (Figure S3B,C). This, along with rigorous prior characterization of its inhibitory profile [25], demonstrated that the antibody could successfully block ADAMTS5 function in the developing embryo, making possible combined ADAMTS1 and ADAMTS5 inactivation (*Adamts5*^X^) during defined temporal windows of cardiac development by administration to *Adamts1*^+/−^ dams mated to *Adamts1*^+/−^ males (Fig. 1B). Embryos from timed pregnancies were obtained to determine the effects of combined inactivation in two distinct developmental windows: from E10.5 to E12.5 and from E12.5 to E16.5, and compared to the effect of a control IgG-injection (hereafter described as control) in dams.

### Combined ADAMTS1 and ADAMTS5 inactivation from E10.5 to E12.5 severely impairs cardiac jelly regression and myocardial compaction

**Fig. 2.**
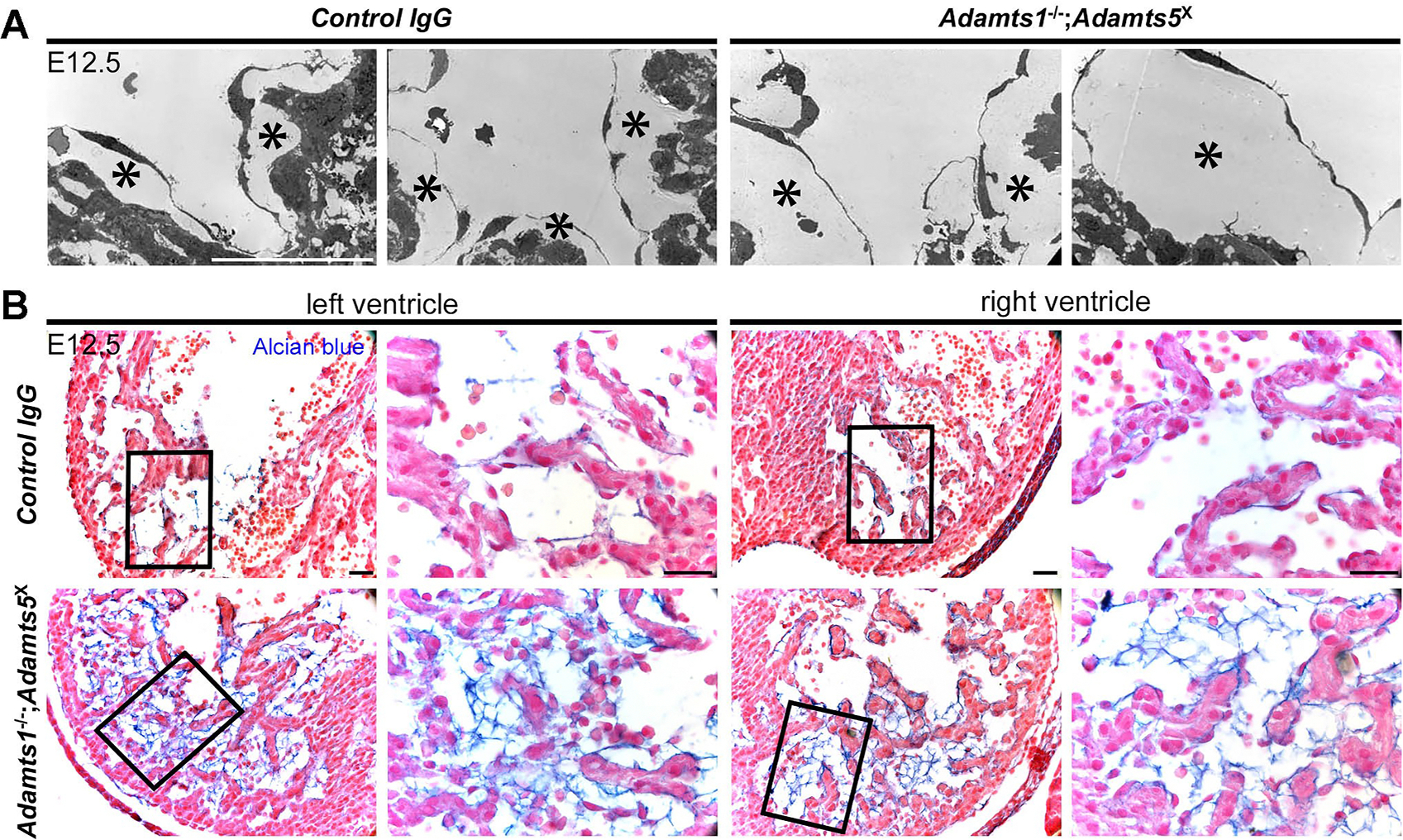
Accumulation of cardiac jelly in *Adamts1*^−/−^;*Adamts5*^X^ hearts. **A.** Transmission electron microscopy images showed accumulated cardiac jelly between the myocardium and endocardium (sub-endocardium, asterisks) in E12.5 *Adamts1*^−/−^;*Adamts5*^X^ hearts, contrasting with hearts from control IgG-injected mice. Images are representative of *N* = 3 each. **B.** Alcian blue-stained sections of ventricular myocardium showed increased proteoglycan staining (blue) in E12.5 *Adamts1*^−/−^;*Adamts5*^X^ hearts. Images are representative of *N* = 4 control and 7 *Adamts1*^−/−^;*Adamts5*^X^ hearts. Scale bar = 50μm.

By E12.5, the myocardium normally has a well-defined compact outer layer with trabeculae projecting into the ventricular lumen. The intimal aspect of each is lined with endocardium and a modest amount of cardiac jelly normally remains between endocardium and myocardium (Fig. 1B). We observed that thickness of the compact myocardium was considerably attenuated in both ventricles of *Adamts1*^−/−^;*Adamts5*^X^ hearts, with fewer, thinner trabeculae and a conspicuous cell-free space between the myocardium and endocardium (Fig. 1B–D) suggestive of cardiac jelly accumulation. Although cardiac jelly also persisted in the *Adamts5*^X^ left ventricular wall, there was no statistically significant alteration in the thickness of the compact myocardium or development of trabeculae (Fig. 1B–D). Electron microscopy performed after tissue fixation in conditions that preserve proteoglycans and their cellular attachments [36], consistently showed massive expansion of cardiac jelly, seen as a ground-glass appearance in *Adamts1*^−/−^;*Adamts5*^X^ left ventricle (Fig. 2A). Aggregates of versican, a known ADAMTS1 and ADAMTS5 substrate, with hyaluronan (HA) are the most abundant component of cardiac jelly (Fig. 2B), although aggrecan is also reported as a minor HA-binding proteoglycan in the mouse heart [7]. However, staining for neither aggrecan nor the ADAMTS cleavage epitope NITEGE in aggrecan was observed in wild type, *Adamts1*^−/−^, *Adamts5*^X^ or *Adamts1*^−/−^;*Adamts5*^X^ hearts with reliable and sensitive antibodies at E12.5 (Figure S4). In contrast to lack of cardiac jelly in *Vcan*^hdf/hdf^ mutant hearts (Figure S5A), there was no visible change in cardiac jelly histologically in *Acan*^cmd/cmd^ mutants, which lack aggrecan, at E9.5. Compact and trabecular myocardium of the *Acan*^cmd/cmd^ mutant was comparable to the wild type littermates at E12.5 and E14.5 (Figure S5B, C). We therefore focused on versican processing in the context of cardiac jelly remodeling by ADAMTS1 and ADAMTS5.

**Fig. 3.**
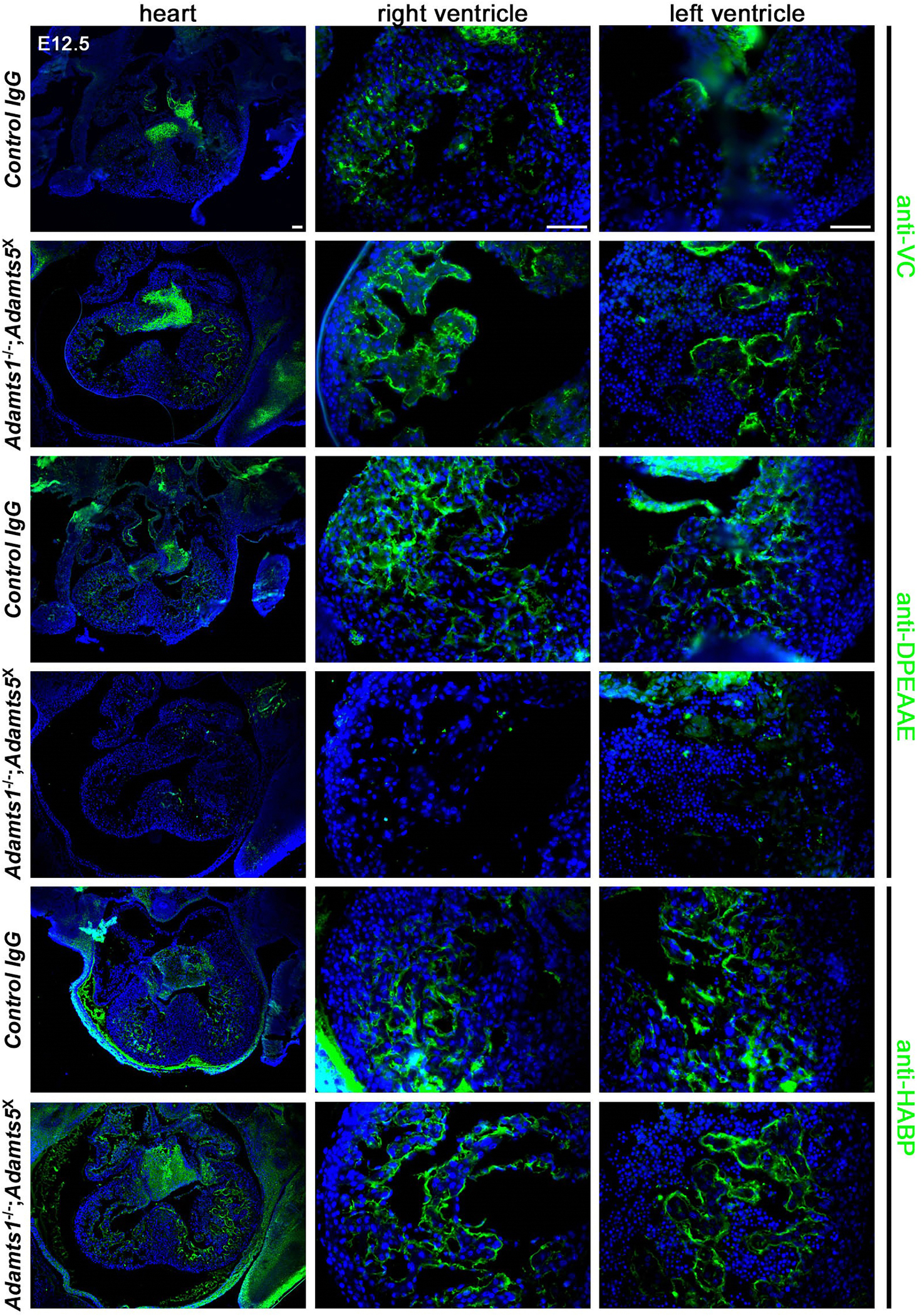
Altered versican dynamics in *Adamts1*^−/−^;*Adamts5*^X^ ventricular myocardium. Immunofluorescence microscopy showed versican accumulation (anti-VC) and lack of ADAMTS-cleaved versican (anti-DPEAAE) in E12.5 *Adamts1*^−/−^;*Adamts5*^X^ hearts, without an apparent change in anti-HABP staining. Images are representative of *N* = 4 control and 7 *Adamts1*^−/−^;*Adamts5*^X^ hearts. Scale bar = 50μm.

Consistent with the high chondroitin sulfate content of versican, stronger alcian blue staining was seen in *Adamts1*^−/−^;*Adamts5*^X^ hearts (Fig. 2B). We observed considerably stronger versican staining in *Adamts1*^−/−^;*Adamts5*^X^ myocardium, and correspondingly, staining with anti-DPEAAE, a neoepitope antibody reacting with ADAMTS cleaved versican [33] was dramatically reduced in the *Adamts1*^−/−^;*Adamts5*^X^ myocardium compared to controls (Fig. 3). In contrast, staining with HA-binding protein (HAbp) suggested that HA abundance was unaltered, although increased staining for cartilage link protein (Crtl1), which stabilizes the interaction between HA and versican and is necessary for cardiac development [9,37], and of fibronectin, which binds to the versican G3 domain [38], were observed (Figure S6).

### A genetically engineered mouse mutant with cleavage-resistant versican (*Vcan*^AA/AA^) has comparable cardiac jelly accumulation as *Adamts1*^−/−^; *Adamts5*^x^ embryos

**Fig. 4.**
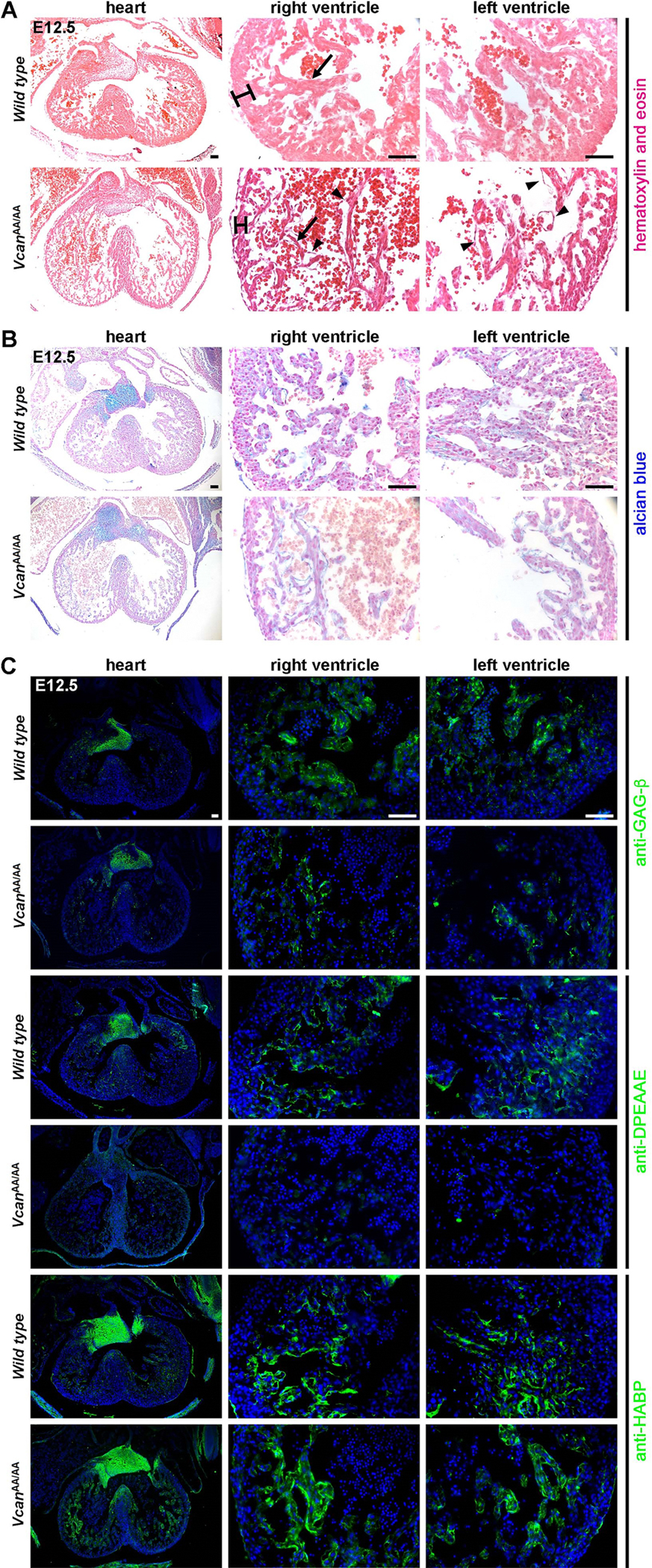
*Vcan*^AA/AA^ hearts have unremodeled cardiac jelly. **A, B.** Hematoxylin and eosin (A) and alcian blue-stained (B) E12.5 *Vcan*^AA/AA^ hearts have thin compact myocardium (brackets), attenuated trabecular myocardium (arrows) and excess cardiac jelly seen as swelling and bubbles (arrowheads) between trabecular myocardium and endocardium. **C.** Reduced GAG-β staining (versican), lack of DPEAAE staining (cleaved versican) and no change in HABP staining in *Vcan*^AA/AA^ hearts compared to wild type. Images are representative of *N* = 6 wild type and 8 *Vcan*^AA/AA^. Scale bar = 50μm.

The *Vcan*^AA^ allele bears mutations that prevent proteolysis at a well-characterized (canonical) site of attack (Glu^441^-Ala^442^) by several ADAMTS proteases [26]. *Vcan*^AA/AA^ mutants survive [23,26], and cardiac development was not previously investigated in these mutants. In E12.5 *Vcan*^AA/AA^ hearts, we observed a thinner compact myocardium and trabeculae than in wild type littermate hearts, resembling in appearance the *Adamts1*^−/−^;*Adamts5*^X^ hearts (Fig. 4A). Accumulation of cardiac jelly was evident in the sub-endocardial space in *Vcan*^AA/AA^ hearts from the greater separation of endocardium and myocardium and by abundant alcian blue-stained cardiac jelly at E12.5 (Fig. 4B). As expected, anti-DPEAAE reactivity was lost in the *Vcan*^AA/AA^ hearts, since the introduced mutations altered the epitope (to DPEAAA); versican staining was also attenuated by the introduced mutations, since they lie within the immunogen sequences used to generate the antibodies anti-VC and anti-GAGβ [26,39] (Fig. 4C). Despite accumulation of cardiac jelly observed at E12.5, alcian blue staining was comparable in *Vcan*^AA/AA^ and control hearts at E16.5 and at birth (Figure S7), indicating that versican remodeling was restored after E12.5, possibly occurring at several other known cleavage sites in the core protein [22].

### Combined ADAMTS1 and ADAMTS5 inactivation after the peak of cardiac jelly regression impairs rotational alignment of the outflow tract and ventricular chambers

**Fig. 5.**
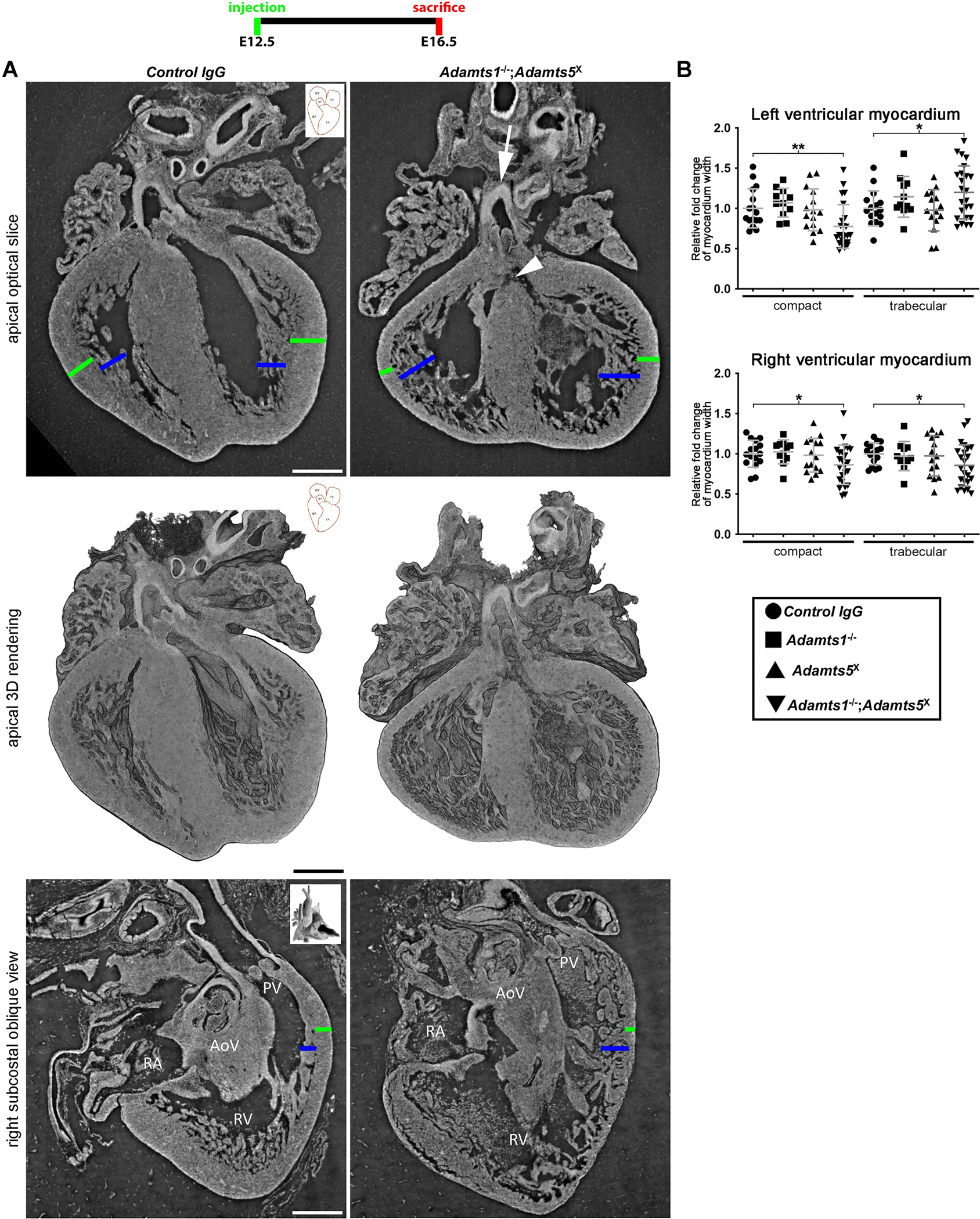
Combined inactivation of ADAMTS1 and ADAMTS5 from E12.5 to E16.5 results in myocardial and cardiac rotational anomalies. Synchrotron imaging in optical slices corresponding to the apical 5-chamber view used in echocardiography and 3D renderings (top and center panels) revealed overriding aorta (arrow), membranous ventricular septal defect (arrowhead), thinned compact myocardium (green bars) and ventricular non-compaction (blue bars) in E16.5 *Adamts1*^−/−^;*Adamts5*^X^ hearts. The right subcostal oblique view (bottom panels) showed right ventricular myocardial anomalies including thinned compact myocardium (green bars) and ventricular non-compaction (blue bars). AoV, aortic valve; PV, pulmonic valve; RA, right atrium; RV, right ventricle. *N* = 4 each group. Scale bar = 300μm. **B.** *Adamts1*^−/−^;*Adamts5*^X^ hearts have thinner left ventricular compact myocardium, increased trabecular myocardium and thinner right ventricle compact and trabecular myocardium compared to the control. *N* ≥ 11. *p ≤ 0.05; **p ≤ 0.01, Student *t*-test. Data points in B show fold change of mean, the error bars show S.E.M.

Synchrotron imaging and histology after combined inactivation from E12.5 to E16.5 showed variable cardiac defects including overriding aorta, membranous ventricular septal defect (VSD) and compact ventricular myocardial thinning along with ventricular non-compaction/hypertrabeculation in the left ventricle of *Adamts1*^−/−^;*Adamts5*^X^ hearts, but not *Adamts1*^−/−^ and *Adamts5*^X^ hearts (Fig. 5, Figure S8, Supplemental videos 1 and 2). Overriding aorta and associated VSD occurred with high penetrance in *Adamts1*^−/−^;*Adamts5*^X^ mutants (Table S1), suggestive of outflow tract malrotation and failure of ventricular septal fusion to the atrioventricular septum. In contrast, *Adamts1*^−/−^, *Adamts5*^X^ and *Vcan*^AA/AA^ hearts did not have VSD or rotational anomaly. Ventricular septal fusion is initiated around E13.5-E14.0 and is completed by E14.5. By E16.5, the mesenchymal tissue (cap) at the basal end of the ventricular septum was compacted in the IgG control-injected hearts and the *Adamts5*^X^ hearts, but not in *Adamts1*^−/−^; *Adamts5*^X^ hearts (Figure S8). Versican staining was comparable in *Adamts1*^−/−^;*Adamts5*^X^ myocardium and control IgG-injected *Adamts1*^−/−^ hearts at E16.5 (Figure S9). Anti-DPEAAE staining was consistently eliminated in *Adamts1*^−/−^;*Adamts5*^X^ hearts (Figure S9), indicating lack of compensatory versican cleavage at the canonical site by other proteases.

### N-terminomics suggests reduced glypican-6 or reduced glypican-6 proteolysis in *Adamts1*^−/−^;*Adamts5*^x^ hearts

**Fig. 6.**
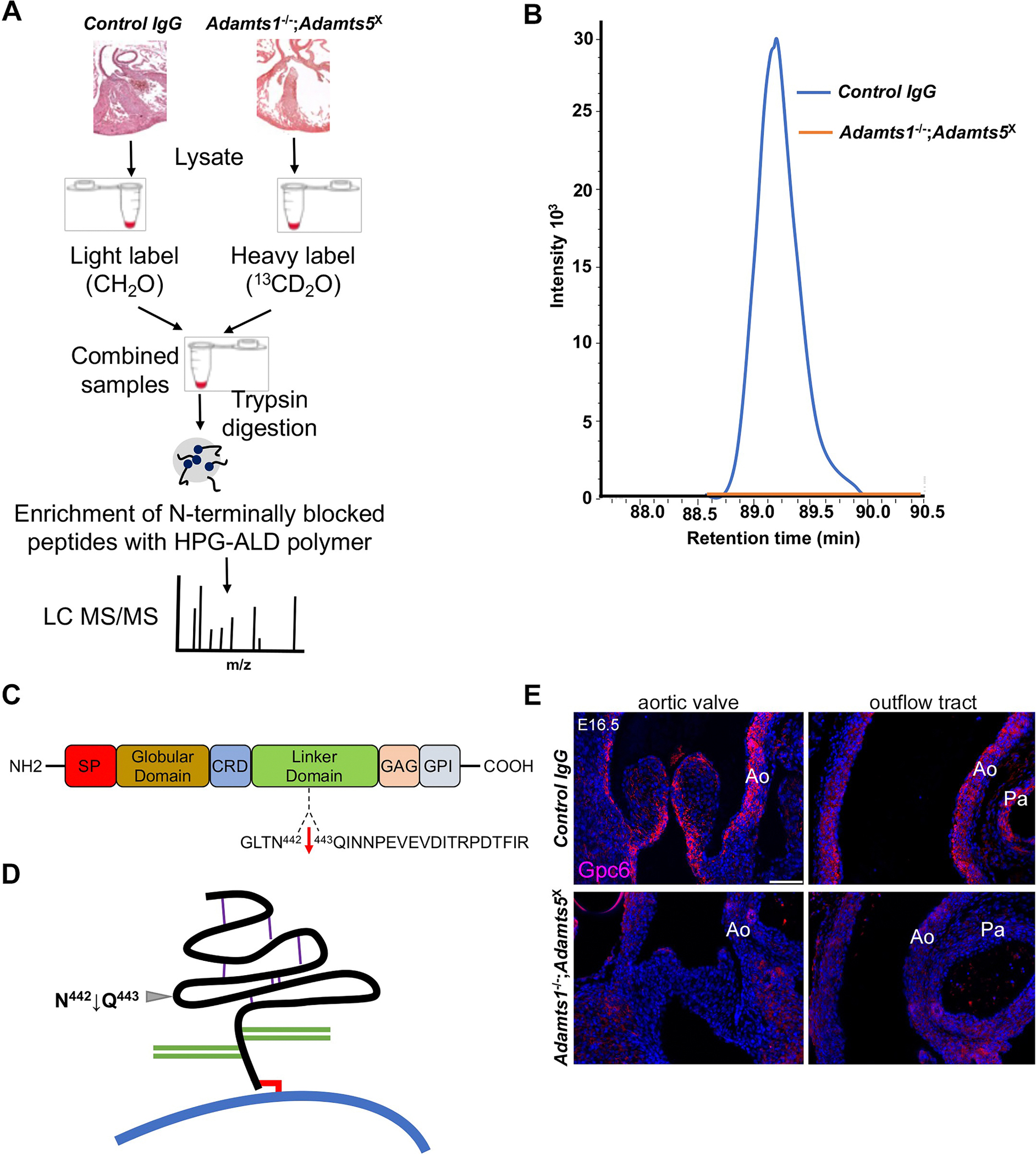
Apparent reduction in glypican-6 cleavage in *Adamts1*^−/−^;*Adamts5*^X^ hearts and identification of the cleavage site using N-terminomics. **A.** Schematic of the N-terminomics approach (TAILS) used for analysis of E16.5 hearts from *Adamts1*^−/−^;*Adamts5*^X^ (*N* = 6) and control embryos (*N* = 6). Hearts of each genotype were pooled and the pooled protein extract was used to obtain technical triplicates of duplex (heavy/light) dimethyl TAILS. **B.** Retention time-aligned extracted ion chromatograms (EICs) showing the light dimethyl-labeled QINNPEVEVDITRPDTFIR peptide in control hearts (blue line) whereas no isotopically heavy dimethyl-labeled peptide (orange line) was detected in *Adamts1*^−/−^;*Adamts5*^X^ hearts. Data is from three replicate TAILS experiments using one set of pooled control vs mutant hearts. **C.** Domain structure of glypican-6 showing the location of the cleaved peptide bond N^442^-Q^443^ in the linker between CRD and GAG domains. **D.** Model of glypican-6 structure and cleavage site. Purple lines indicate disulfide bonds, the red line shows the GPI-anchor. GAG chains are shown as green lines, the cell membrane as a thick curved blue line. **E**. Immunofluorescence showed no glypican-6 staining in *Adamts1*^−/−^;*Adamts5*^X^ E16.5 hearts. Ao, aorta; Pa, pulmonary artery. Images are representative of *N* = 3 in each group. Scale bar = 50μm.

Since the observed rotational anomalies in *Adamts1*^−/−^;*Adamts5*^X^ hearts were not seen in *Vcan*^AA/AA^ hearts, we considered an alternative impact of ADAMTS1 and ADAMTS5. Substrates were sought by unbiased quantitative comparison of protein N-termini from *Adamts1*^−/−^; *Adamts5*^X^ hearts vs control hearts (injected with an isotype-matched, non-inhibitory IgG), using the N-terminomics strategy Terminal Amine Isotopic Labeling of Substrates (TAILS) [40] (Fig. 6A). 450 differentially abundant N-terminally labeled/blocked peptides were identified, of which 317 were positionally internal peptides suggesting a proteolytic origin, but only a few of these arose from secreted/ECM proteins (Table S2). Among peptides less abundant in the *Adamts1*^−/−^;*Adamts5*^X^ heart degradome of secreted/ECM molecules, we identified an internal, N-terminally labeled peptide from the linker region of the GPI-anchored cell-surface proteoglycan glypican-6 only in control hearts (Fig. 6B,C). The peptide N-terminus was non-tryptic, confirming, together with its N-terminal dimethyl label, that the peptide arose by proteolysis of glypican-6 rather than trypsin digestion of the extracted proteins during preparation for mass spectrometry. Glypicans are evolutionarily conserved cell surface heparan sulfate (HS) proteoglycans linked to the cell membrane via a glycosylphosphatidylinositol (GPI) anchor [41]. The predicted cleavage site identified by the peptide, Asn^442^-Gln^443^ is in a region termed the linker which is upstream of the HS chains and flanked by predicted disulfide bonds (Fig. 6D).

Because of a predicted disulfide bond formed by cysteines flanking Asn^442^-Gln^443^, this cleavage event alone is unlikely to lead to fragmentation or shedding of the N-terminal region of glypican-6 (Fig. 6D). Application of TAILS to the medium of cells co-expressing ADAMTS1 or ADAMTS5 with glypican-6 after co-transfection identified additional glypican-6 cleavages but not peptides indicating cleavage at Asn^442^-Gln^443^ (Figure S10A,B, Tables S3,S4). Although neither in vitro experiment produced peptides identified the Asn^442^-Gln^443^ site found in the heart, possibly because of the different samples and mass spectrometers used, they indicated that glypican-6 cleavage occurred in the presence of both proteases. N-terminomics analysis also revealed cleavages in each protease and identified additional putative ADAMTS1 and ADAMTS5 substrates including secreted/ECM and cell-surface/transmembrane molecules (Tables S3,S4)

Because the lack of the cleaved peptide in *Adamts1*^−/−^;*Adamts5*^X^ hearts could also reflect reduced glypican-6 abundance, we used glypican-6 immunostaining, which showed considerable reduction in the outflow tract of *Adamts1*^−/−^;*Adamts5*^X^ hearts (Fig. 6E). Since *Gpc6* expression and function during cardiac development was previously undescribed, we determined *Gpc6* mRNA distribution at E12.5 and E16.5 in wild type embryo hearts, localizing it in the smooth muscle layer of the outflow tract, with weaker signal in the adventitia, while absent in the endothelium, which overlapped with *Adamts1* and *Adamts5* expression at these time points (Figure S11A). *Gpc6* mRNA was also localized in outflow tract valves, epicardium and myocardium (Figure S11A). RNAscope suggested less intense *Gpc6* signal in the *Adamts1*^−/−^;*Adamts5*^X^ outflow tract (Figure S11A). However, RT-qPCR showed comparable *Gpc6* mRNA levels in *Adamts1*^−/−^;*Adamts5*^X^ and control hearts, possibly because this analysis used RNA extracted from the entire heart rather than just the outflow tract (Figure S11B).

### *Gpc6*^−/−^; mouse embryos have cardiac rotational defects resembling those of *Adamts1*^−/−^;*Adamts5*^x^ embryos

**Fig. 7.**
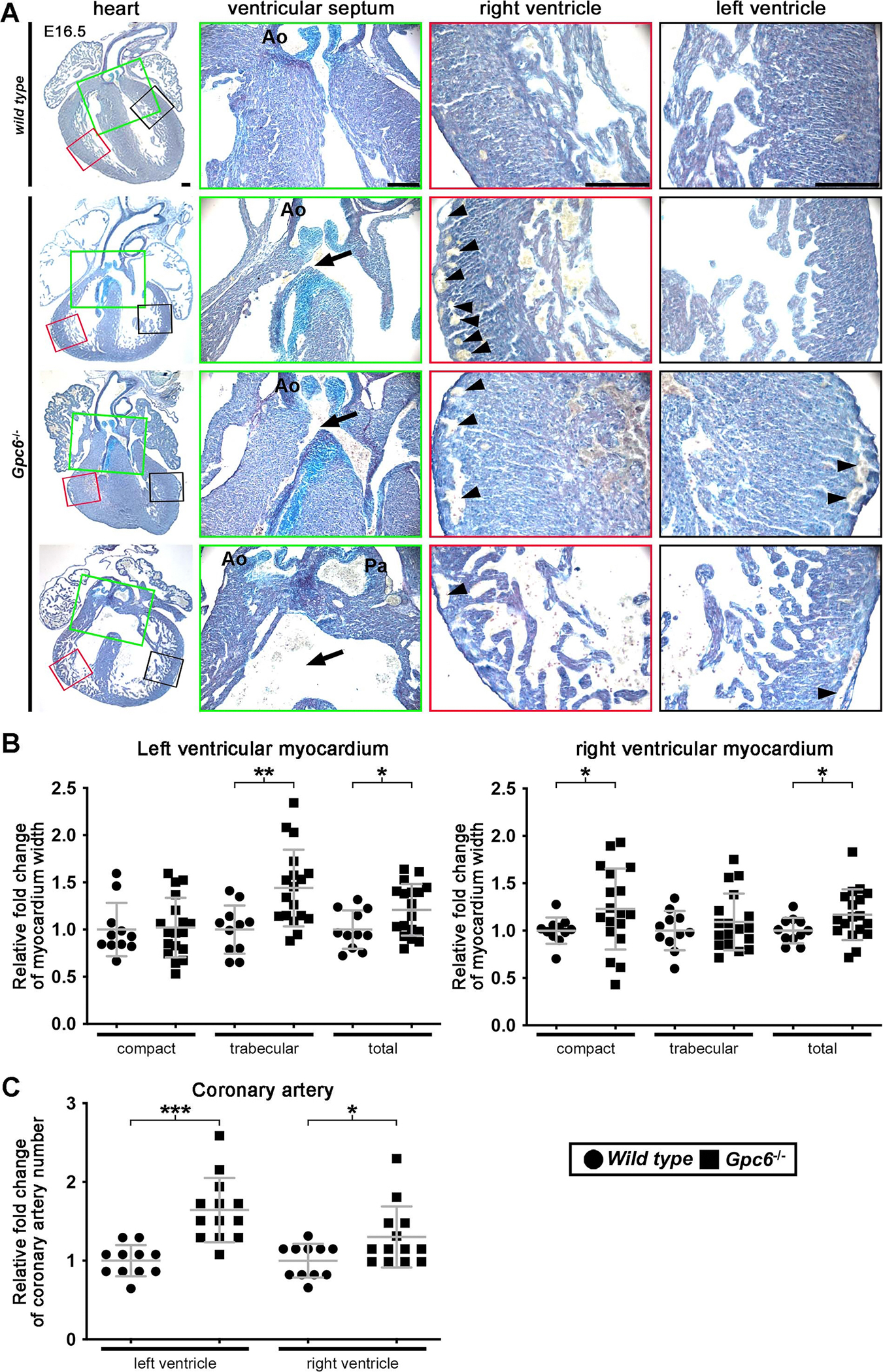
*Gpc6*^−/−^; embryos have diverse congenital heart defects including those seen in *Adamts1*^−/−^;*Adamts5*^X^ hearts. **A.** RGB stained *Gpc6*^−/−^; hearts exhibit congenital heart malformations that resemble those of *Adamts1*^−/−^;*Adamts5*^X^ hearts including ventricular septal defect (arrows) and overriding aorta. Arrowheads show the more numerous sub-pericardial coronary vessels in *Gpc*6^−/−^; hearts. Ao, aorta; Pa, pulmonary artery. Images are representative of *N* = 11 *Gpc6*^−/−^; hearts and *N* = 18 *Adamts1*^−/−^;*Adamts5*^X^ hearts. Scale bar = 100μm. **B.** Increased trabecular and increased compact and total left and right ventricular myocardium in E16.5 *Gpc6*^−/−^; hearts. **C.** Increased number of coronary vessels in E16.5 *Gpc6*^−/−^; hearts. *n* ≥ 11. *p ≤ 0.05; **p ≤ 0.01; ***p ≤ 0.001. Student *t*-test. Data in panels B and C are shown as fold change of mean, the error bars show S.D, p values were determined by the Student *t*-test.

To determine whether the rotational anomalies observed in *Adamts1*^−/−^;*Adamts5*^X^ hearts resulted from reduced Gpc6, we evaluated *Gpc6* knockout and wild type littermate hearts histologically at E16.5. *Gpc6*^−/−^; embryos consistently showed a spectrum of cardiac anomalies resembling those of *Adamts1*^−/−^;*Adamts5*^X^ hearts, including VSD and overriding aorta, as well as double outlet right ventricle, which was not seen in *Adamts1*^−/−^;*Adamts5*^X^ hearts (Fig. 7A, Table S5). *Gpc6*^−/−^; myocardium showed variable myocardial thinning and ventricular non-compaction, similar to *Adamts1*^−/−^;*Adamts5*^X^ hearts, as well as thickened ventricular compact myocardium in places. Due to the variation in ventricular wall thickness of the *Gpc6*^−/−^; hearts, they were not statistically different from wild type hearts (Fig. 7B). Sub-pericardial coronary vessels were more frequently seen in E16.5 *Gpc6*^−/−^; hearts than in wild type littermate hearts, suggesting aberrant coronary vascular development (Fig. 7C). There was no change in proteoglycan content in the *Gpc6*^−/−^; cardiac jelly at E12.5 compared to littermate controls (Figure S12).

**Fig. 8.**
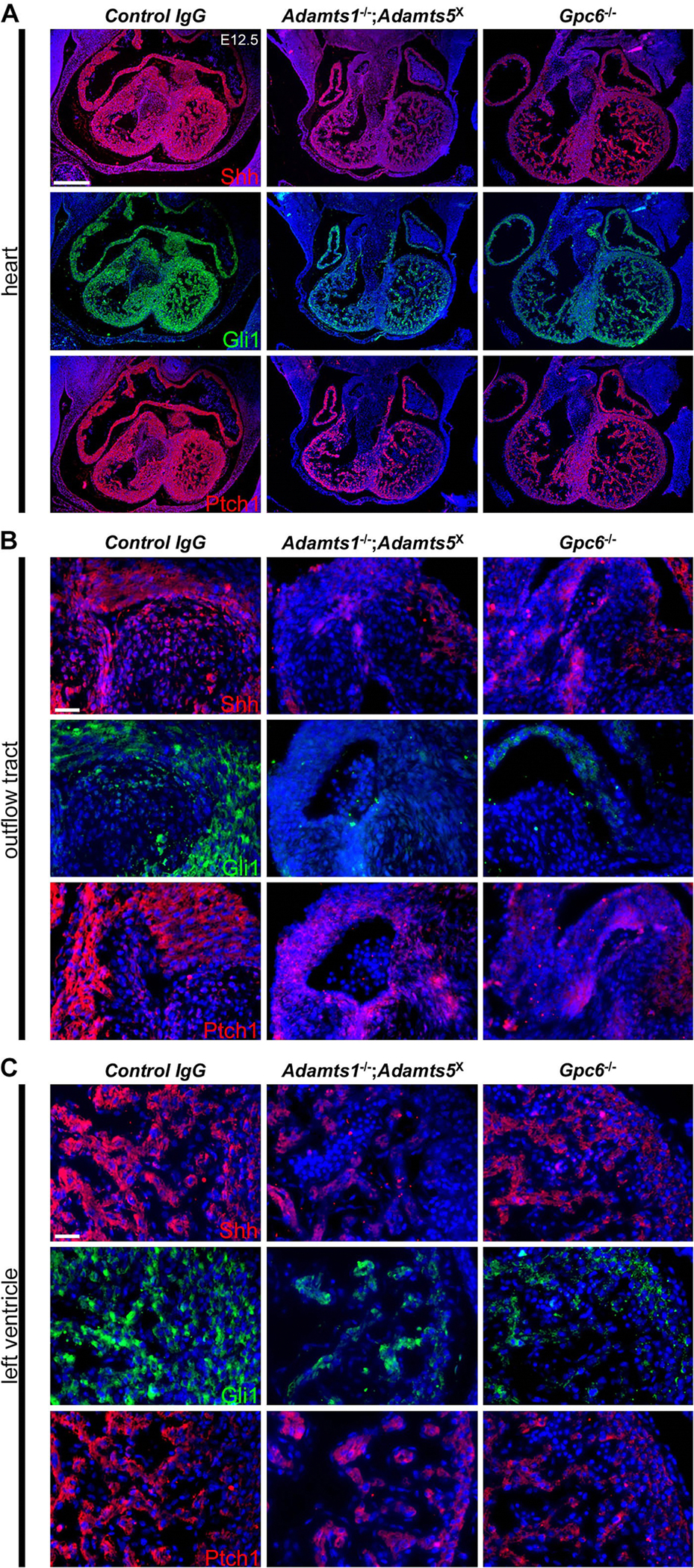
Reduced staining of hedgehog pathway proteins in E12.5 *Adamts1*^−/−^;*Adamts5*^X^ and *Gpc6*^−/−^; hearts. Immunofluorescence staining showed reduced Shh, Gli1 and Ptch1 staining in an overview of the heart (A), outflow tract (B), and ventricular myocardium (C) in *Adamts1*^−/−^;*Adamts5*^X^ and *Gpc6*-deficient E12.5 hearts. Images are representative of *N* = 4 control, 6 *Adamts1*^−/−^;*Adamts5*^X^ and 5 *Gpc6*^−/−^;. Scale bar = 200μm (A); 25μm (B,C).

Since prior work identified a role for *Gpc6* in regulation of Shh signaling during skeletal and intestinal development [42–44], we measured mRNA levels of Shh target genes *Shh, Ptch1, Gli1* and *Gli2* in E12.5 *Adamts1*^−/−^;*Adamts5*^X^ hearts and found reduction of each mRNA (Figure S13A). RNAscope showed less intense signal for these mRNAs in the outflow tract and ventricular myocardium of *Gpc6* knockouts as well as in E12.5 *Adamts1*^−/−^;*Adamts5*^X^ hearts (Figure S13B). Immunofluorescence demonstrated reduced Shh, Ptch1 and Gli1 staining in the outflow tract and ventricular myocardium of *Adamts1*^−/−^;*Adamts5*^X^ and *Gpc6*^−/−^; hearts (Fig. 8).

## Discussion

Combined deletion of closely related genes with potentially redundant roles is needed to detect overlapping functions, in this case, in proteolysis, since an intact homolog can potentially buffer the missing activity. Combined deletion additionally unmasks the impact of transcriptional adaptation, by which genetic robustness is maintained in the face of inactivating mutations [29,45], and was reported within the ADAMTS family [46]. Curiously, although *Adamts5* expression was unchanged in *Adamts1* knockouts we observed *Adamts1* upregulation in response to administration of the function blocking ADAMTS5 antibody, which is presently unexplained. This finding, together with their shared activity profile against proteoglycan substrates and their observed coexpression in the developing heart suggested a possible undervaluation of ADAMTS5 in cardiac development. In addition to expression by the same cell types (valve interstitial cells, endocardium and cardiomyocytes), ADAMTS1 and ADAMTS5 are secreted and thus, can also act non-cell autonomously to modify the pericellular and interstitial ECM in the outflow tract. The present work shows that combined ADAMTS1 and ADAMTS5 inactivation consistently resulted in additional severe anomalies not observed in either single mutant.

The combined genetic-pharmacologic knockout approach, which was not previously used in the protease or cardiovascular research fields, offers a novel solution for combinatorial deletion when genetic models are unavailable, or the coding genes tightly linked. It offers the advantage for functional inactivation in discrete temporal developmental windows, which was the approach used here. The combined genetic-pharmacologic strategy is appropriate for future combined deletion of two other tightly linked ADAMTS genes, *Adamts8* and *Adamts15*, whose functions remain to be fully elucidated, both individually and in combination and are of interest since *Adamts8* is implicated in pulmonary hypertension [47]. Genetic-pharmacologic inactivation may be preferable to constitutive genetic deletion, which affects all subsequent developmental events, making it challenging to determine the extent to which late developmental processes are affected by antecedent events. This problem is currently bypassed by inducible conditional or global deletion of genes, which have shortcomings including incomplete gene deletion and potential toxicity of tamoxifen.

Three lines of evidence indicated that the observed myocardial defects in *Adamts1*^−/−^;*Adamts5*^X^ hearts before E12.5 resulted from a requirement of both proteases for versican proteolysis, i.e., dramatic accumulation of versican-rich cardiac jelly, reduced staining with anti-DPEAAE, and observation of cardiac jelly accumulation in mice with versican resistant to cleavage. Cardiac jelly is histologically invisible unless specifically sought by staining of glycosaminoglycans, and since it is highly hydrated, it can be under-estimated because of dehydration during processing for paraffin embedding. It is best visualized by ultrastructural methods that preserve cell-ECM interactions, which we employed. During early ventricular development, cardiac jelly forms a thick acellular layer in the sub-endocardial space, and may generate signals that drive cardiomyocyte proliferation and differentiation [7]. Indeed, versican core protein and chondroitin sulfate chains bind several morphogens and growth factors including Ihh, TGFβ and VEGF-A [13, 48]. Upon initiation of myocardium compaction, cardiac jelly persists in bubble-like remnants tethered to the compact myocardium and trabeculae continue to mature in these bubbles [7]. After trabecular extension is complete, cardiac jelly disappears, but the precise mechanisms of this were undefined. We show that ADAMTS1 and ADAMTS5, which jointly cleave at the Glu^441^-Ala^442^ site and at additional shared or individually preferred sites [22], are essential for cardiac jelly clearance. The findings also suggest that cardiac jelly persistence beyond its developmentally regulated clearance is detrimental to subsequent proper development of compact and trabecular myocardium. The absence of DPEAAE staining, indicative of versican cleavage, in *Adamts1*^−/−^; *Adamts5*^X^ hearts, suggested that ADAMTS1 and ADAMTS5 are primarily responsible for versican cleavage at this site in the cardiac jelly. Alternatively, a proteolytic fragment of versican may be essential for proper cardiomyogenesis. Indeed, the occurrence of morphologically similar myocardial anomalies in *Vcan*^AA/AA^ mice suggests a role for versikine, the N-terminal product of cleavage at the Glu^441^-Ala^442^ site for cardiomyogenesis, since the *Vcan*^AA/AA^ mice have only a single uncleavable site, yet ADAMTS1 and ADAMTS5 cleave efficiently at additional sites in the core protein. Although cleavage of additional ADAMTS1 and ADAMTS5 substrates is likely also reduced in *Adamts1*^−/−^;*Adamts5*^X^ hearts, the similarity of the myocardial anomaly in *Adamts1*^−/−^; *Adamts5*^X^ and *Vcan*^AA/AA^ mice suggests that impaired versican Glu^441^-Ala^442^ site cleavage in cardiac jelly is a major contributor. We did not observe significant immunostaining of the ADAMTS1/ADAMTS5 substrate aggrecan or aggrecan neoepitope in the developing mouse heart and embryonic hearts from *Acan*^cmd/cmd^ mutant mice did not have defective cardiac jelly formation or compact and trabecular myocardium. Thus, ADAMTS1 and ADAMTS5 proteolysis of versican, not aggrecan, appears to be specifically crucial for proper cardiomyogenesis. Among versican-degrading ADAMTS proteases, ADAMTS9 was previously shown to be essential for proper cardiac development [49], whereas cardiac anomalies were not reported in ADAMTS4, ADAMTS15 or ADAMTS20 mutant mice. ADAMTS1, ADAMTS4 and ADAMTS5 each cleave the versican core protein at multiple sites [22], providing a potential explanation of lack of continued cardiac jelly accumulation in later development in both *Adamts1*^−/−^;*Adamts5*^X^ and *Vcan*^AA/AA^ mice, where other versicanases could compensate.

*Vcan*^AA/AA^ mice lacked rotational anomalies, suggesting that these were unrelated to persistent cardiac jelly. Therefore, TAILS was used as an unbiased strategy to compare E16.5 *Adamts1*^−/−^;*Adamts5*^X^ and control hearts for identification of proteolytic peptides more abundant in the latter. TAILS identified an internal glypican-6 peptide with a non-tryptic N-terminus in the controls that was absent in *Adamts1*^−/−^; *Adamts5*^X^ hearts. Glypican-6 is required for skeletal and intestinal development and is implicated in heart failure, which results in its upregulation [42,44,50]. Previously, adenoviral overexpression of glypican-6 in cultured cardiomyocytes was shown to increase protein synthesis and induce hypertrophy and heart failure signature genes [50], suggesting glypican-6 as a relevant ADAMTS1 and ADAMTS5 target in cardiac development.

Whereas TAILS suggested reduced glypican-6 proteolysis, its orthogonal evaluation by immunostaining demonstrated reduced abundance in *Adamts1*^−/−^;*Adamts5*^X^ hearts, contrary to the expectation that it would accumulate in the absence of proteolysis, and RNAscope indicated reduced outflow tract *Gpc6* expression after ADAMTS1 and ADAMTS5 inactivation. In addition to being a terminomics method, TAILS is a *de facto* fractionation method and improves the prospect of detecting less abundant proteins in a complex mixture such as whole heart lysate and alternatively, the proteolytically cleaved peptide may be reporting differential glypican-6 abundance. However, since the observed peptide was clearly internal, the lack of glypican-6 staining suggested that glypican-6 cleavage by ADAMTS1 and/or ADAMTS5 could be a stabilizing event that protects against additional degradation or its removal from the cell-surface by internalization, as reported for glypican-3 [51]. A targeted TAILS approach to specifically interrogate glypican-6 proteolysis by ADAMTS1 and ADAMTS5 individually in vitro by co-culture of glypican-6 and ADAMTS1/ADAMTS5 expressing cells, demonstrated cleavage in the presence of each protease, but whether this is a direct action of ADAMTS1 and ADAMTS5 or occurs indirectly via activation of another protease remains to be determined.

Although *Gpc6*^−/−^; mice die at birth, their cardiac development was not previously investigated [42]. *Gpc6*^−/−^; hearts consistently showed ventricular septal defects and a spectrum of rotational anomalies, including overriding aorta and double outlet right ventricle which are likely to be incompatible with survival. The dramatic reduction of glypican-6 after ADAMTS1 and ADAMTS5 inactivation suggested that rotational anomalies in *Adamts1*^−/−^;*Adamts5*^X^ hearts likely result from reduction of glypican-6. Prior work showed that glypican-6 promotes hedgehog signaling during long bone development and intestinal elongation [42–44]. Glypican-6 similarly appears to have a role in Hh signaling during cardiac development, since expression of key genes in the Hh signaling pathway was reduced in *Gpc6*^−/−^; hearts. Importantly, and consistent with a potential role for ADAMTS1 and ADAMTS5 in supporting glypican-6 activity, *Adamts1*^−/−^;*Adamts5*^X^ hearts also showed a reduction in Hh signaling genes.

Notably, Hedgehog signaling was previously investigated in the heart only to a limited extent. Shh was previously not thought to be expressed in the heart, but rather, to be produced by endoderm to influence second heart field and neural crest cells migration during early heart development, atrial septation and outflow tract development [52–54]. The current findings unequivocally show expression of Shh within the heart itself, moreover at later developmental stages. Notably, lack of Shh results in a phenotype resembling Tetralogy of Fallot [55], consistent with the observed anomalies in *Gpc6*^−/−^; and *Adamts1*^−/−^; *Adamts5*^X^ hearts. It is relevant that ADAMTS9 and ADAMTS20 are essential for hedgehog signaling during embryogenesis as a result of their requirement for forming a primary cilium. Experimental evaluation of ADAMTS1 and ADAMTS5 in ADAMTS9- and ADAMTS20-lacking RPE-1 cells, which do not form cilia, had previously excluded a role for ADAMTS1 and ADAMTS5 in ciliogenesis [56]. Thus, the present work uncovers a distinct mode of Shh signaling regulation by ADAMTS1 and ADAMTS5, acting via glypican-6.

In conclusion, the present work, which used an unconventional approach to inactivate two tightly linked protease genes within defined temporal windows, illustrates that ADAMTS1 and ADAMTS5 are together essential for cardiac development and participate in distinct developmental processes. First, they are required for cardiac jelly regression during cardiomyogenesis, where they appear to act primarily via versican processing at the canonical Glu^441^-Ala^442^ site which could be a rate limiting step at the peak of cardiac jelly clearance. It is also possible that the resulting N-terminal fragment, versikine [23], is essential for myocardiogenesis. Later in heart development, ADAMTS1 and ADAMTS5 are required for completion of ventricular septal fusion to the atrioventricular cushions and proper alignment of the outflow tract with the ventricular chambers. These roles appears to be mechanistically dependent on glypican-6, whose presence, in turn, is essential for Hh signaling during the process. Although the cooperative functions of ADAMTS1 and ADAMTS5 are supported by independent genetic experiments embodied in the *Vcan*^AA/AA^ and *Gpc6*^−/−^; mutants, the precise mechanism(s) by which ADAMTS1 and ADAMTS5 affect the abundance of glypican-6 remains to be elucidated.

## Experimental procedures

### Transgenic mice and experimental procedures

Mice hemizygous for an inactivated *Adamts1* allele (*Adamts1*tm^1Dgen^, Deltagen_T1228, MGI: 5,427,602, referred to here as *Adamts*^1+*/*−^;) were rederived by the Case Western Reserve University Transgenic Core from frozen embryos purchased from the Mutant Mouse Regional Resource Centre (Medical Research Council, Harwell, Didcot, United Kingdom) and crossed for 8 generations with wild type C57BL/6 mice (Jackson Laboratories, Bar Harbor, ME). ADAMTS5 inhibitory antibody GSK12F4.1H7 and control IgG2c (each 20 mg/kg in 250 μl of sterile PBS) [25] were delivered by intraperitoneal injection into pregnant wild type or *Adamts1*^+/−^; females. *Vcan*^AA^, *Acan*^cmd/+^and *Gpc6* mutant mice were previously described and were maintained in the C57BL/6 background [26,57,58]. All procedures were approved by the Institutional Animal Care and Use Committee of the Cleveland Clinic (IACUC protocol 2021–2761) or Sunnybrook Research Institute, University of Toronto (IACUC protocol #22,024).

### Synchrotron imaging

PFA-fixed, paraffin-embedded hearts were imaged with synchrotron-based phase-contrast micro-computed tomography at the TOMCAT beamline of the Swiss Light Source (Paul Scherrer Institut, Villigen, Switzerland) applying previously described experimental settings [59]. Phase-contrast was achieved with free space propagation. The x-ray beam was monochromatized to an energy of 21 keV. The samples were placed on a rotating stage in-between source and detector, with a sample-to-detector distance of 19 cm in order to get optimal propagation. The detecting system, consisting of a 20-μm thick LuAG:Ce scintillator, a 4X magnifying objective and a sCMOS detector, resulted in a 4.2 × 3.5 mm^2^ field-of-view with an effective pixel size of 1.63 × 1.63 μm^2^. A full tomographic scan consisted of a 180° rotation of the sample, during which 1801 projection images were acquired at regular intervals. Each projection had an acquisition time of 80 ms, resulting in a total scan time of about 2.4 min per sample. Following acquisition, phase-retrieval was performed using Paganin’s method (δ = 3.7 × 10^−8^, β =1.7 × 10^−10^) [60]. The volumes were then reconstructed from the projections by using the gridrec algorithm [60]. The obtained data were volumes of 2560 × 2560 × 2160 pixels, with a 16-bit pixel depth. The data were then visualized with a combination of Fiji [61] and Amira (Thermo Fisher Scientific) software.

### RNAscope in situ hybridization and β-gal staining

RNA in situ hybridization was performed using RNAscope (Advanced Cell Diagnostics) as previously described [46,62]. Briefly, 6 μm sections were deparaffinized and hybridized to mouse *Adamts1, Adamts5, Gpc6, Shh, Ptch1, Gli1* or *Gli2* probe sets (catalog nos. 463,361, 427,621, 442, 841, 314,361, 402,811, 311,001, or 405,771, respectively; Advanced Cell Diagnostics) using a HybEZ™ oven (Advanced Cell Diagnostics) and the RNAscope 2.5 HD Detection Reagent Kit (322,360; Advanced Cell Diagnostics). In addition, *Adamts1*^+/−^; hearts were stained for β-gal activity as previously described [63]. Briefly, tissue was fixed in 0.25 % glutaraldehyde, washed in rinse buffer (1 M sodium phosphate, 200 mM MgCl_2_, 1 % sodium deoxycholate, 2 % NP-40) placed in staining solution (5 mM potassium ferricyanide, 5 mM potassium ferrocyanide, 20 mg/mL X-Gal in rinse buffer) overnight at 37 °C in the dark. Stained tissue was post-fixed in 4 % paraformaldehyde overnight at 4 °C, processed, embedded in paraffin and 10 μm sections were dehydrated and counterstained with nuclear fast red.

### Real-Time quantitative PCR (RT-qPCR)

RT-qPCR was performed as previously described [46]. Briefly, total RNA was isolated using TRIzol (15596018, Invitrogen), and 1 μg of RNA was reverse transcribed with SuperScript III CellsDirect cDNA synthesis system (46–6321, Invitrogen, Thousand Oaks, CA). Power SYBR Green Mastermix (4367659, Applied Biosystems, Foster City, CA) was used for RT-qPCR using an Applied Biosystems 7500 instrument. *Gapdh* served as the housekeeping gene. The ΔΔCt method was applied to calculate relative mRNA expression levels of target genes using GraphPad Prism. Supplemental Table 6 provides the primer sequences.

### Histology and immunofluorescence microscopy

Hearts were submerged in ice-cold PBS supplemented with 1 M KCl for 5 min to induce cardioplegia, fixed in 4 % PFA, embedded in paraffin and 6 μm sections were obtained. Hematoxylin and eosin, RGB-trichrome, and alcian blue staining were performed as previously described [64,65]. Myocardial thickness was measured from a fixed point halfway between the base and apex of the heart in the plane of the aortic-mitral valve continuity. Antigen retrieval was used prior to immunostaining by microwaving for 4 × 1.5 min in citrate buffer (10 mM sodium citrate, 2 mM EDTA, 0.05 % (v/v) Tween-20, pH 6.2). Supplemental Table 7 lists the antibodies used. Alexa fluor 488- or 568-conjugated goat anti-mouse, rabbit, or goat immunoglobulin (Molecular Probes, Invitrogen, Carlsbad CA) was used as the secondary antibodies and nuclei were counterstained and mounted with Vectashield-DAPI mounting medium (Vector Labs, Burlingame, CA). Images were obtained using an Olympus BX51 microscope with Leica DFC 7000T camera and Leica Application Suite V4.6 software (Leica, Wetzlar, Germany). Coronary vessels were counted from heart sections having visible aortic and mitral valve continuity in the section for consistency and identified by blood-filled lumena in the ventricular myocardium that did not communicate with the ventricular chamber.

### Transmission electron microscopy (TEM)

The left ventricular wall was fixed in 2.5 % glutaraldehyde plus 4 % paraformaldehyde in 0.2 M sodium cacodylate buffer, pH 7.4 containing 0.07 % ruthenium hexamine trichloride to preserve the proteoglycan-cell interface [66]. After fixation at 4 °C overnight, tissue blocks were washed twice with 0.2 M sodium cacodylate buffer, pH 7.4, processed in propylene oxide/eponate12 medium at room temperature, embedded in pure Eponate 12 and polymerized overnight. 85 nm thick sections were stained with osmium tetroxide and viewed with a PhillipsCM12/STEM transmission electron microscope (FEI Company, Hillsboro, OR) equipped with a digital 11-megapixel CCD camera (Gatan, Pleasanton, CA).

### Cell culture and transfection

HEK293F cells were transfected (PEI MAX, 24765, Polysciences) with plasmids encoding either human ADAMTS1, catalytically inactive human ADAMTS1 with Glu^402^ mutated to Ala (ADAMTS1 EA, control), human ADAMTS5 or an empty plasmid vector (as control). Separately, the HEK293F cells were transfected with a human GPC6 expression plasmid and these cells were co-plated with ADAMTS/control expressing cells in a 1:1 ratio in 10 cm dishes in Dulbecco’s Modified Eagle Medium (DMEM) supplemented with 10 % fetal bovine serum (FBS), 100 U/ml penicillin, and 100 μg/ml streptomycin at 37 °C in a 5 % CO_2_ humidified chamber and cultured overnight. After overnight incubation, the cells were cultured in serum-free, indicator-free medium for 48 h and the medium was collected. The cells were washed with PBS prior to incubation with phosphatidylinositol-specific phospholipase C (2.5 U/ml; catalog no. P6466, ThermoFisher) for 1 h at 37 °C in phenol-free, serum-free DMEM to release GPC6 from the cell membrane and added to the collected medium for N-terminomics [67].

### Terminal amine isotopic labeling of substrates (TAILS) of mouse heart

TAILS [40] was done essentially as recently described [68]. Whole hearts from *Adamts1*^−/−^;*Adamts5*^X^ (*N* = 6) and control embryos (*N* = 6) were obtained at E16.5 by cesarian section, washed with PBS, diced and washed to minimize blood content. Hearts from each genotype were pooled and technical triplicates from the pools were used for duplex dimethyl TAILS. Tissue was sonicated in a water bath at 70 % amplitude in T-PER (ThermoFisher Scientific, catalog no. 78,510) with addition of 1X protease inhibitors (cOmplete protease inhibitor cocktail, cat no. 4693159001; Roche), centrifuged at 8000 × g for 10 min and the supernatant was collected in a new tube. The pellets were sonicated in 6 M guanidine hydrochloride (GuHCl), supplemented with 1X protease inhibitors and incubated for 16 h at 4 °C. 1 μl of benzonase was added to degrade DNA/RNA in the samples and incubated for 10 min on ice. Samples were centrifuged at 8000 × g and the supernatant of this extraction was pooled with the T-PER extract. Proteins were isolated by methanol-chloroform precipitation and the protein pellets were reconstituted in a minimum volume of 6 M GuHCl and pH was adjusted with 100 mM NaOH and maintained with 100 mM HEPES buffer pH 7.5. Total protein was measured using the Bradford assay and 200 μg of protein was taken for TAILS sample preparations. Samples were reduced with 5 mM dithiothreitol (DTT) final by incubating at 60 °C for 1 h, followed by alkylation with 15 mM iodoacetamide (IAA) for 30 min in dark at room temperature. The reaction was quenched by adding a final concentration of 10 mM DTT and incubating for 30 min at 37 °C. Knockout and control samples were labeled separately with stable isotopic tags by adding 40 mM final concentration of heavy or light formaldehyde respectively in the fume hood, sodium cyanoborohydride was added immediately at 20 mM final concentration, adjusted to pH 6–7 and samples were incubated at 37 °C for 16 h. Fresh formaldehyde (20 mM final), along with a final concentration of 10 mM sodium cyanoborohydride were added and incubated for 3 h. The reaction was quenched with 100 mM (final concentration) Tris pH 6.8 and incubated at 37 °C for 1 h. The pH was adjusted to 8 using 1 M HEPES. The isotopically labeled samples were mixed and digested overnight with trypsin at a 1:50 (trypsin:protein) ratio. 20 μg of these peptides were retained as the preTAILS sample for mass spectrometry. The remaining peptide mixture was incubated with hyperbranched polyglycerol-aldehydes (HPG-ALD, Flintbox, https://www.flintbox.com/public/project/1948/) at a 5:1 polymer:protein ratio [40]. The mixture was filtered through 10 kDa cut-off filters (EMD Millipore) and unbound peptides representing the N-terminally blocked peptide population were collected in the flow-through (TAILS samples). TAILS and preTAILS peptides were desalted on a Sep-Pac (Waters), vacuum-centrifuged until dry and re-suspended in 1 % acetic acid.

### TAILS of cell culture supernatant

Proteins were isolated from 10 ml of serum-free, indicator-free medium by methanol-chloroform precipitation, the pellets were reconstituted in a minimum volume of 6 M GuHCl and pH was adjusted with 100 mM NaOH and maintained with 100 mM HEPES buffer pH 7.5. Labeling of proteins, trypsin digestion and enrichment of N-terminally labeled peptides by HPG-ALD polymer was done as above.

### Mass spectrometry

The peptides from TAILS of the heart were analyzed on a Thermo-Fisher Scientific Fusion Lumos tribrid mass spectrometer interfaced with a Thermo Ultimate 3000 nano-UHPLC. The HPLC column was a Dionex 15 cm × 75 μm id Acclaim Pepmap C18, 2 μm, 100 Å reversed phase capillary chromatography column. 5 μL volumes of the sample were injected, peptides were eluted from the column by an acetonitrile/0.1 % formic acid gradient at a flow rate of 0.3 μL/min and introduced in-line into the mass spectrometer over a 120-minute gradient. The nanospray ion source was operated at 1.9 kV. The digest was analyzed using a data-dependent method with 35 % collision-induced dissociation fragmentation of the most abundant peptides every 3 s and an isolation window of 0.7 *m/z* for ion-trap MS/MS. Scans were conducted at a maximum resolution of 120,000 for full MS. Dynamic exclusion was enabled with a repeat count of 1 and ions within 10 ppm of the fragmented mass were excluded for 60 s.

Mass spectrometer .raw files from TAILS analysis of the hearts were imported into Proteome Discoverer 2.2 (PD 2.2) (Thermo Fisher Scientific). Analysis parameters were set as precursor mass tolerance of 10 ppm, and fragment mass tolerance of 0.6 Da. Static modification was set as carbamidomethyl (C), whereas dynamic modifications included light (+28.031 Da)/heavy (+34.063 Da) dimethyl adduct at N-terminus/K, oxidation (M, P), deamidation (N, R), acetylation (N-terminal), and N-terminal Gln-to pyro-Glu. Spectra were searched using a mouse database from UniProt with isoforms. A false discovery rate (FDR) was calculated by creating a decoy database using percolator node from PD 2.2 and a strict cutoff was applied at 1 % FDR. Peptide groups from PD2.2 were exported as separate Excel files for preTAILS and TAILS samples and combined manually [69]. Statistical analysis was performed using command line stand alone version of CLIPPER V1. CLIPPER merges multiple collision-induced dissociations (CIDs) and different oxidation, amidation and charge states per peptide and calculates intensity-weighted ratios of abundances in protease-treated and control samples. Intensities are also used to assess the relative quality of quantification in the form of a quantification confidence factor (QCF) [70]. N termini are automatically annotated for their position in mature proteins, and natural N termini are extracted to determine a ratio cutoff for neoN termini generated by the protease of interest [71].

Peptides from TAILS of culture medium were analyzed using a Bruker TimsTof Pro2 Q-Tof mass spectrometry system operating in positive ion mode, coupled with a CaptiveSpray ion source (both from Bruker Daltonik GmbH, Bremen). The HPLC column was a Bruker 15 cm × 75 μm id C18 ReproSil AQ, 1.9 μm, 120 Å reversed-phase capillary chromatography column. One μL volumes of the extract were injected and the peptides eluted from the column by an acetonitrile/0.1 % formic acid gradient at a flow rate of 0.3 μL/min were introduced into the source of the mass spectrometer on-line. The digests were analyzed using a Parallel Accumulation–Serial Fragmentation (PASEF) DDA method to select precursor ions for fragmentation with a TIMS-MS scan followed by 10 PASEF MS/MS scans. The TIMS-MS survey scan was acquired between 0.60 and 1.6 Vs/cm2 and 100–1700 *m/z* with a ramp time of 166 ms. The total cycle time for the PASEF scans was 1.2 s and the MS/MS experiments were performed with collision energies from 20 eV (0.6 Vs.cm2) to 59 eV (1.6 Vs/cm2). Precursors with 2–5 charges were selected with the target value set to 20,000 a.u and intensity threshold to 2500 a.u. Precursors were dynamically excluded for 0.4 s. Bruker TimsTof Pro2 Q-Tof mass spectrometry .d files were imported into FragPipe19.0 and searched against Homo sapiens database (Uniprot ID: UP000005640) with 42,390 protein sequences including isoforms. A decoy database was generated and added to the database. MSFragger −3.7, IonQuant-1.8.10 and Percolator were integrated into FragPipe. Analysis parameters in MSFragger were set as precursor mass tolerance of 10 ppm, and fragment mass tolerance of 0.6 Da. Static modification was set as carbamidomethyl (C), whereas dynamic modifications included light (+28.031 Da) or heavy (+34.063 Da) dimethyl adduct at N-terminus/K, oxidation (M, P), deamidation (N, R), acetylation (N-terminal), and N-terminal Gln-to pyro-Glu). Spectra were validated using percolator with 0.5 minimum probability at both the peptide and protein level. Quantification was performed using IonQuant for the labeling. Intensities were normalized across the runs, and only unique and razor peptides were used for quantification. The data was imported into Perseus 1.6.5.0 for visualization and statistical analysis. Peptides modified at their N-termini and having quantitative values were retained and positionally mapped on the respective protein sequence using TopFIND 3.0 (https://topfind.clip.msl.ubc.ca/topfinder) [69]. All N-terminally labeled/blocked peptides other than those presenting the protein start/methionine removed or resulting from signal peptide/-propeptide/transit peptide processing were considered as “internal,” i. e., potentially proteolytically cleaved peptides.

#### Availability of proteomics data:

The mass spectrometry proteomics data have been deposited to the ProteomeXchange Consortium via the PRIDE [72] partner repository with the dataset identifiers PXD045706 (data from orbitrap Fusion Lumos experiments) and PXD045868 (data from Bruker Tims2 ToF Pro2 experiments).

A description of antibodies and primers can be found in the manuscript supplement.

## Supplementary Material

supplementary

video 1

video 2

## Data Availability

Data will be made available on request.
