# Supplementary material for: Combined genetic-pharmacologic inactivation of tightly linked ADAMTS proteases in temporally specific windows uncovers distinct roles for versican proteolysis and glypican-6 in cardiac development
Source: Matrix Biol. Author manuscript; Available in PMC 2024 Oct 31. (PMC11526477; doi:10.1016/j.matbio.2024.05.003)
Supplement: supplementary [file NIHMS2028434-supplement-supplementary.pdf]

SUPPLEMENTAL MATERIAL

Combined genetic-pharmacologic inactivation of tightly linked ADAMTS proteases in temporally specific windows uncovers distinct roles for versican proteolysis and glypican-6 in cardiac development

Timothy J. Mead, Sumit Bhutada, Simon J. Foulcer, Niccolò Peruzzi, Courtney Nelson, Deborah E. Seifert, Jonathan Larkin, Karin Tran-Lundmark, Jorge Filmus, Suneel S. Apte

1) Supplemental Figures (S1-S13) with Supplemental Figure Legends
2) Supplemental Tables S1-S7
3) Legends for Supplemental Videos 1-2
4) Supplemental Videos 1-2

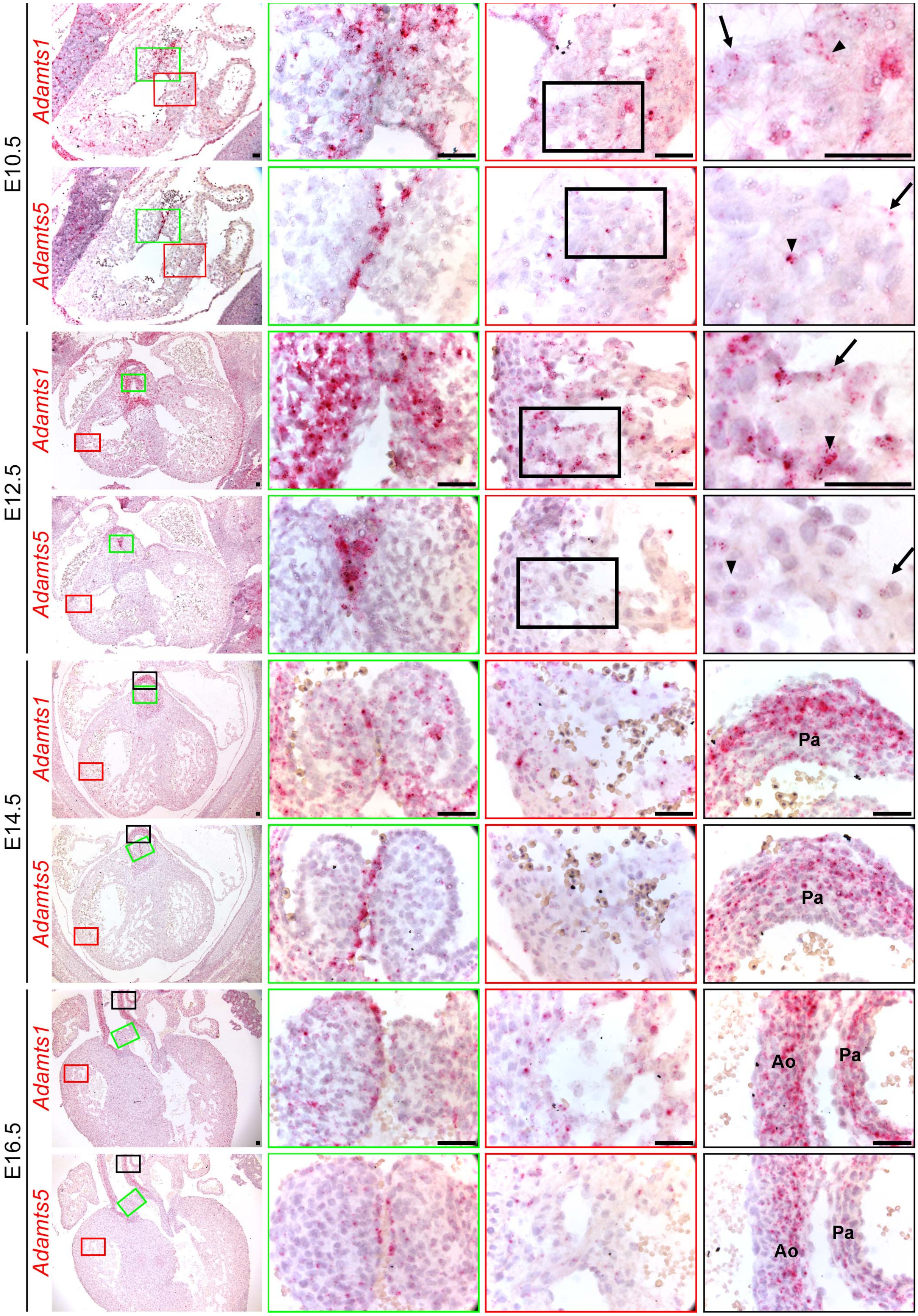

E10.5
Adamts1
Adamts5
E12.5
Adamts1
Adamts5
E14.5
Adamts1
Pa
Adamts5
Pa
E16.5
Adamts1
Ao
Pa
Adamts5
Ao
Pa

Figure S1. *Adamts1* and *Adamts5* are expressed in the outflow tract and myocardium during mouse cardiac development. RNAscope in situ hybridization showing expression (red) of *Adamts1* and *Adamts5* in the cardiac outflow tract and heart valves as well as endocardium (arrows) and myocardium (arrowheads) from E10.5 through E16.5. Nuclei are counterstained with hematoxylin (blue). Ao, aorta; Pa, pulmonary artery. Images are representative of N= 3. Scale bar = 25μm.

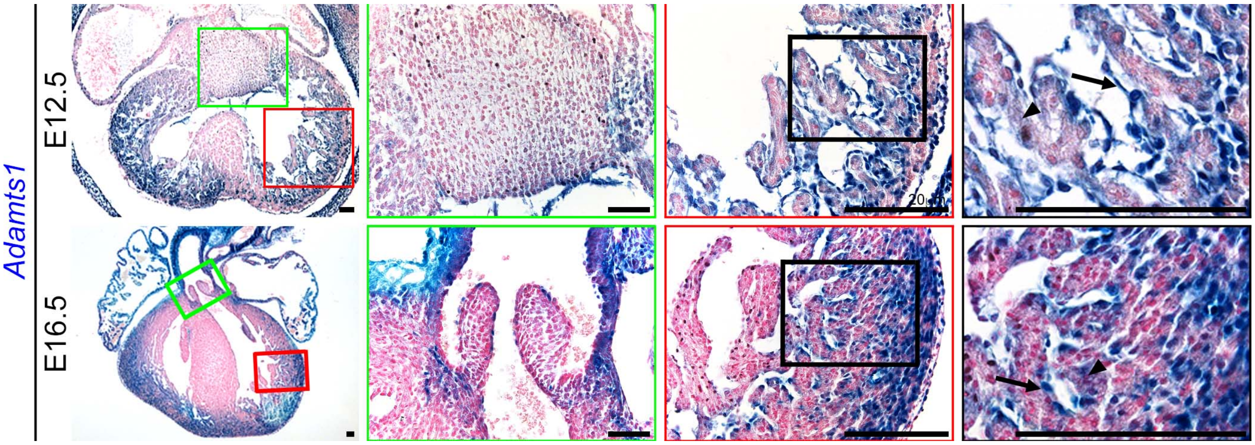

Figure S2. *Adamts1* is expressed in the outflow tract and myocardium during mouse cardiac development. β-galactosidase staining showed *Adamts1* expression (blue) in E12.5 and E16.5 *Adamts1*$^{+/-}$ hearts. In addition to the outflow tract and heart valves, expression is also seen in endocardium (arrows) and myocardium (arrowheads). Images are representative of N=5 at each time point. Scale bar = 50μm.

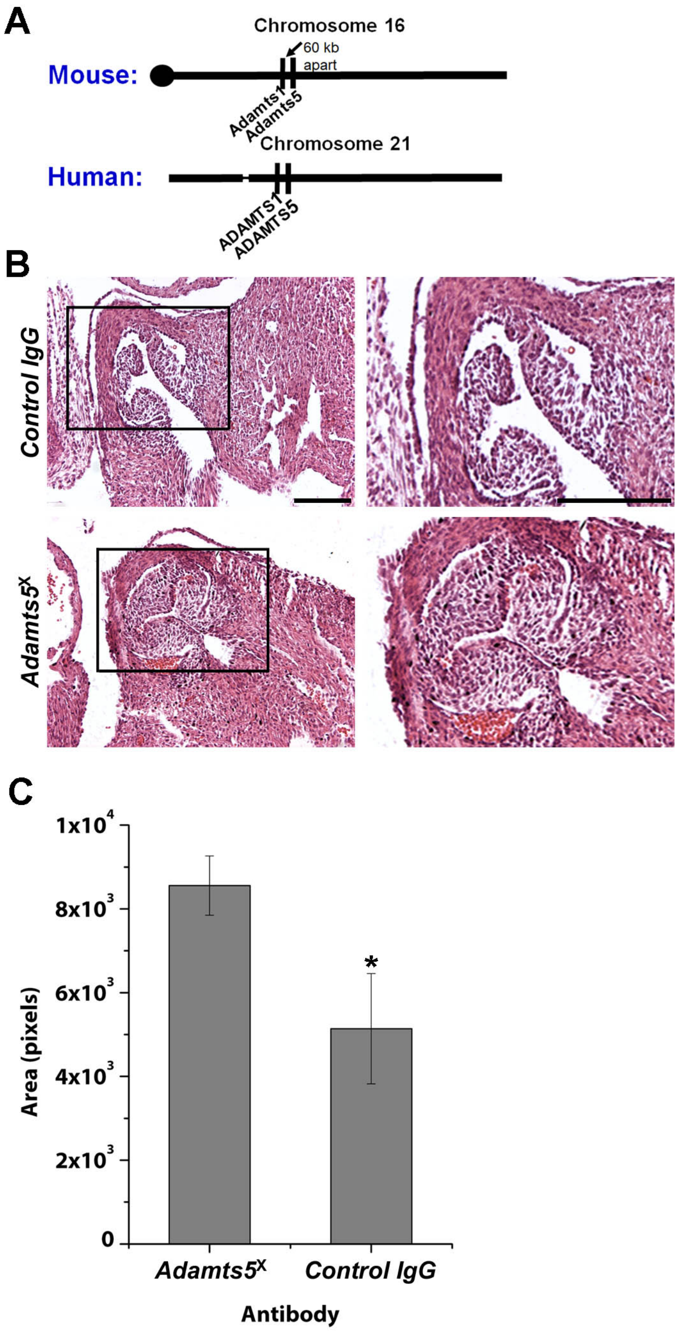

Figure S3. Pharmacological inactivation of ADAMTS5 recapitulates pulmonic stenosis observed in *Adamts5*$^{-/-}$ mice. A. Cartoon depicting tight linkage of *Adamts1* and *Adamts5* loci in mouse and human genomes. **B.** Hematoxylin-eosin staining showed enlarged pulmonic valve leaflets at E16.5 in GSK12F4.1H7-treated hearts (bottom panel; top panel shows control IgG

treated). Images are representative of N=6 in each group. **C.** Quantification of the leaflet size is shown on the right. n=3; $p<0.05$. Scale bar = 100μm.

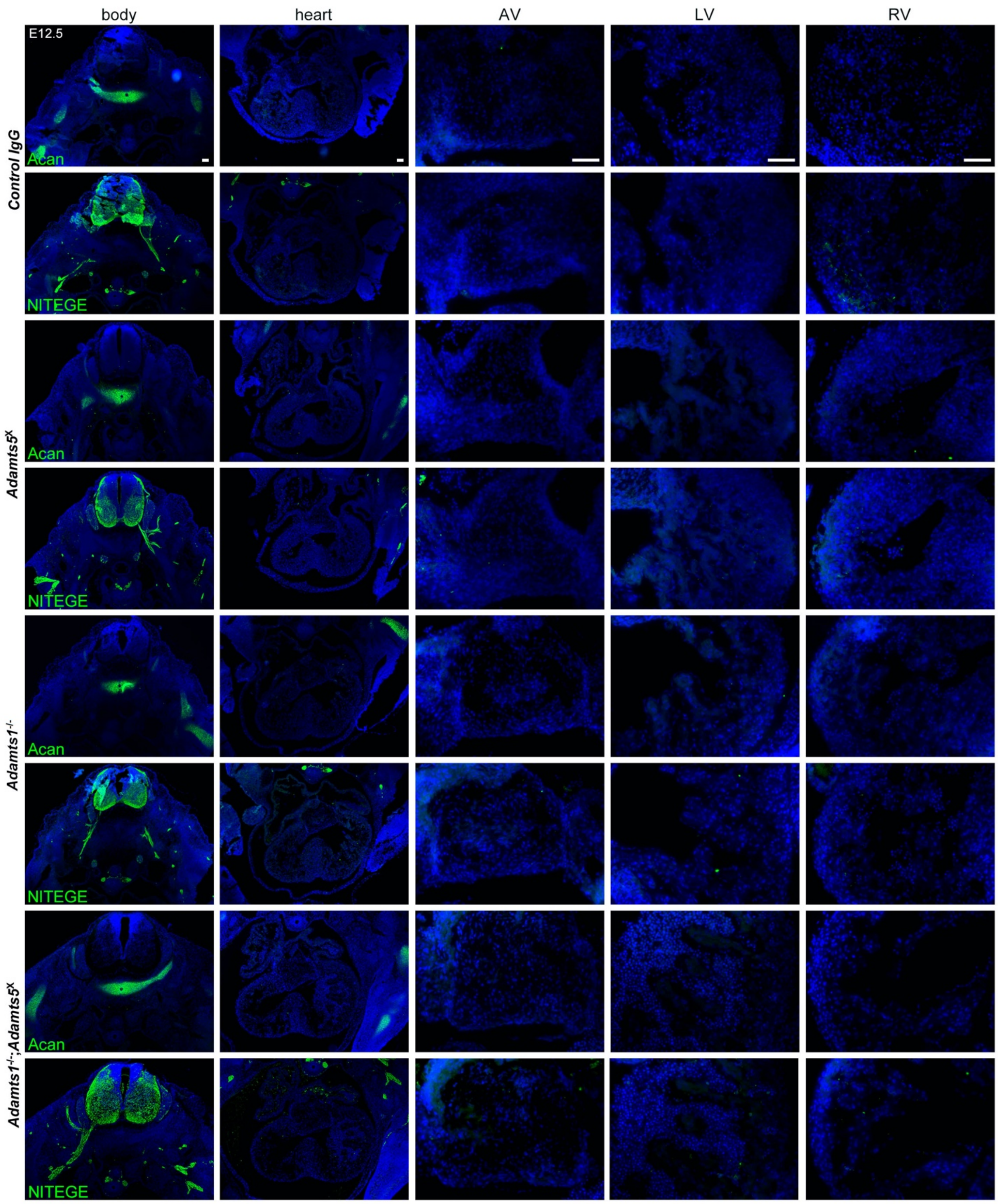
body
heart
AV
LV
RV
E12.5
Control IgG
Adamts5^x
Adamts1^-/-
Adamts1^-/-;Adamts5^x
Acan
NITEGE

Figure S4. No change in aggrecan staining in *Adamts1$^{-/-}$*;*Adamts5^{X}* hearts. Aggrecan staining was seen in cartilage and NITEGE staining (cleaved aggrecan) was evident in the spinal cord of E12.5 *Adamts1$^{-/-}$*, *Adamts5^{X}* and *Adamts1$^{-/-}$*;*Adamts5^{X}* embryos. However, no aggrecan or NITEGE staining was seen in the heart. Images are representative of N= 3 for each group. Scale bar = 50μm.

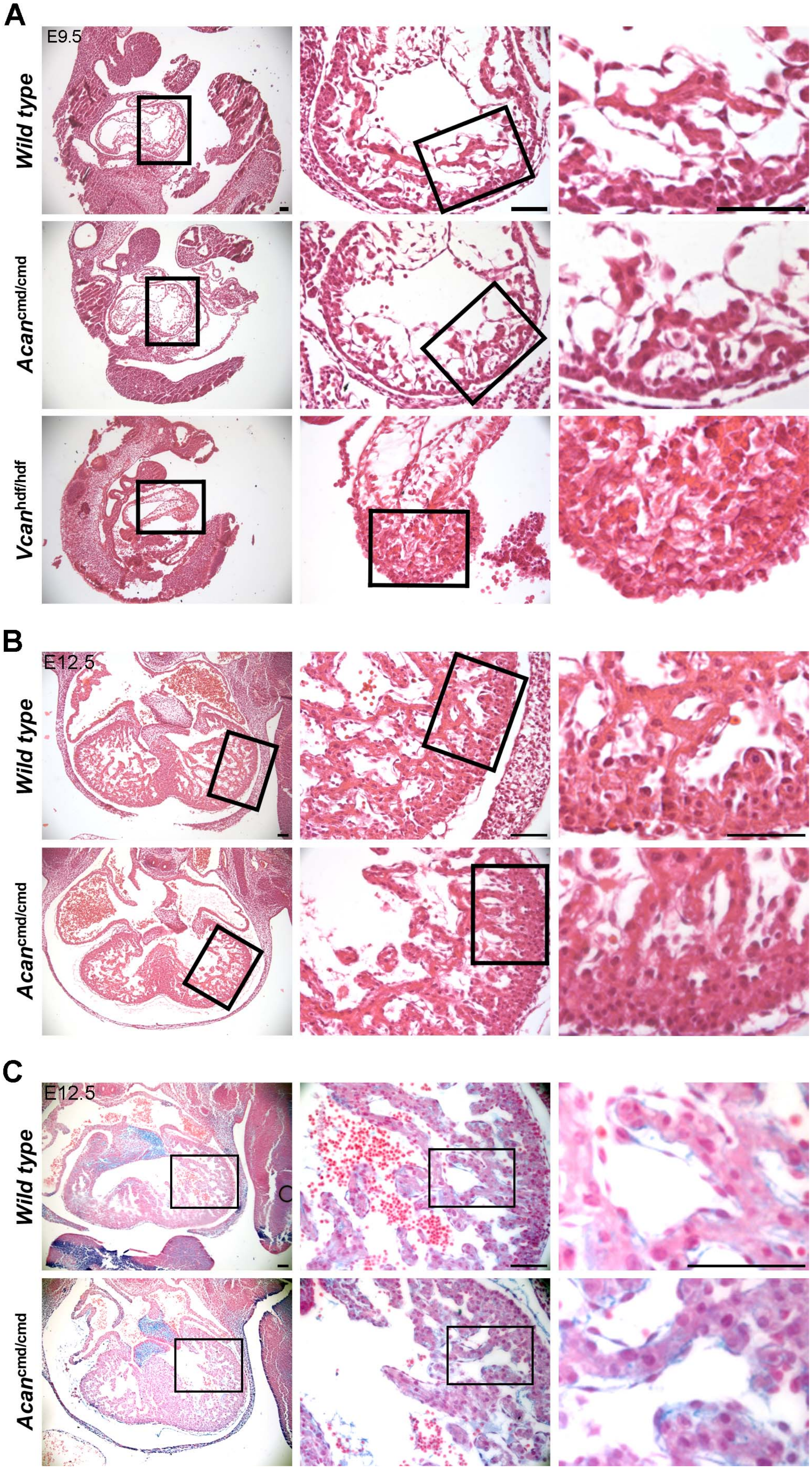
A
E9.5
Wild type
Acancmd/cmd
Vcanhdf/hdf
B
E12.5
Wild type
Acancmd/cmd
C
E12.5
Wild type
Acancmd/cmd

Figure S5. *Acan*-deficient myocardium develops normally. A. Hematoxylin and eosin-stained E9.5 *Acan*$^{cmd/cmd}$ hearts have normal myocardial structure and growth comparable to wild-type littermate whereas *Vcan*$^{hdf/hdf}$ hearts lack cardiac jelly. Images are representative of N=4, 6 and 3 in the control, *Acan*$^{cmd/cmd}$ and *Vcan*$^{hdf/hdf}$, respectively. **B.** Hematoxylin and eosin-stained E12.5 *Acan*$^{cmd/cmd}$ hearts have similar myocardial structure as wild type littermates. **C.** Alcian blue-stained E12.5 *Acan*$^{cmd/cmd}$ hearts showed cardiac jelly comparable to wild type littermate. Scale bar = 50μm. Boxes in the left-hand two panels indicate regions shown at higher magnification in the panels to their right.

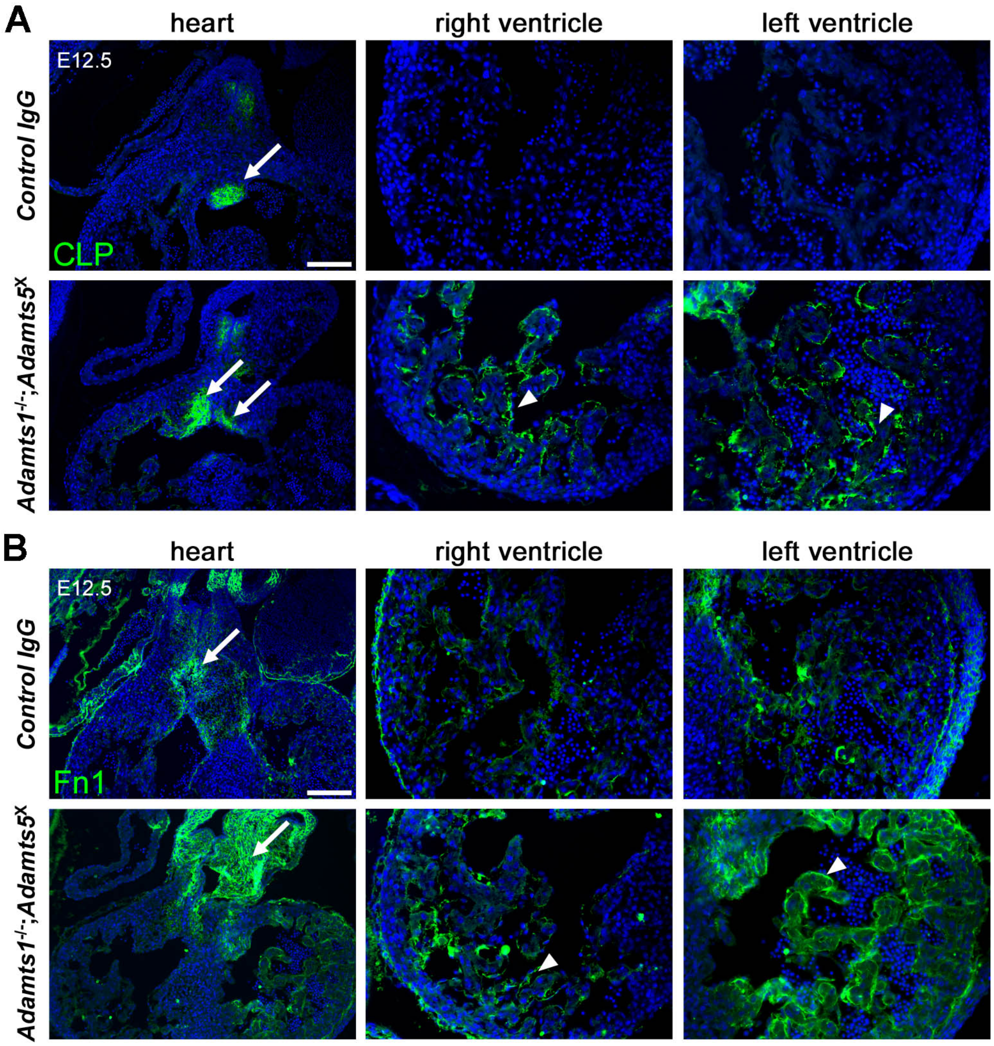

Figure S6. Enhanced cartilage link protein and fibronectin staining in *Adamts1$^{-/-}$*;*Adamts5^{X}* hearts. A. Staining of cartilage link protein (CLP) is enhanced in valve interstitial cells (arrows) and anomalously present in ventricular endocardium (arrowheads) in E12.5 *Adamts1$^{-/-}$*;*Adamts5^{X}* hearts, likely in association with cardiac jelly. **B.** Fibronectin (Fn1) staining is enhanced in outflow tract mesenchyme (arrows) and trabecular myocardium (arrowheads) of *Adamts1$^{-/-}$*;*Adamts5^{X}* hearts. Images are representative of N=3 for each group. Scale bar = 50μm.

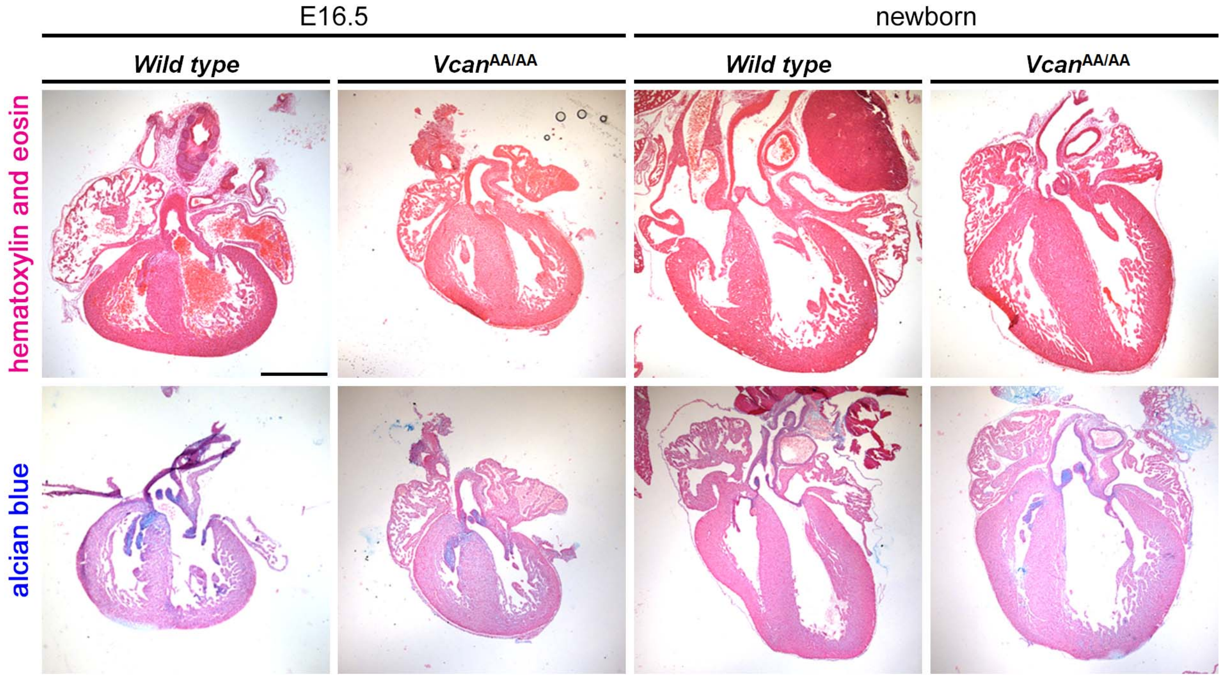

Figure S7. Normal heart architecture in late embryonic and newborn *Vcan*$^{AA/AA}$ hearts. Hematoxylin- eosin and Alcian blue stained E16.5 and newborn *Vcan*$^{AA/AA}$ hearts show no anomalies in the outflow tract, ventricular septum or ventricular myocardium and comparable proteoglycan staining as wild type hearts (Alcian blue). Images are representative of N= 5 for wild-type and and N=6 for *Vcan*$^{AA/AA}$, respectively. Scale bar = 50μm.

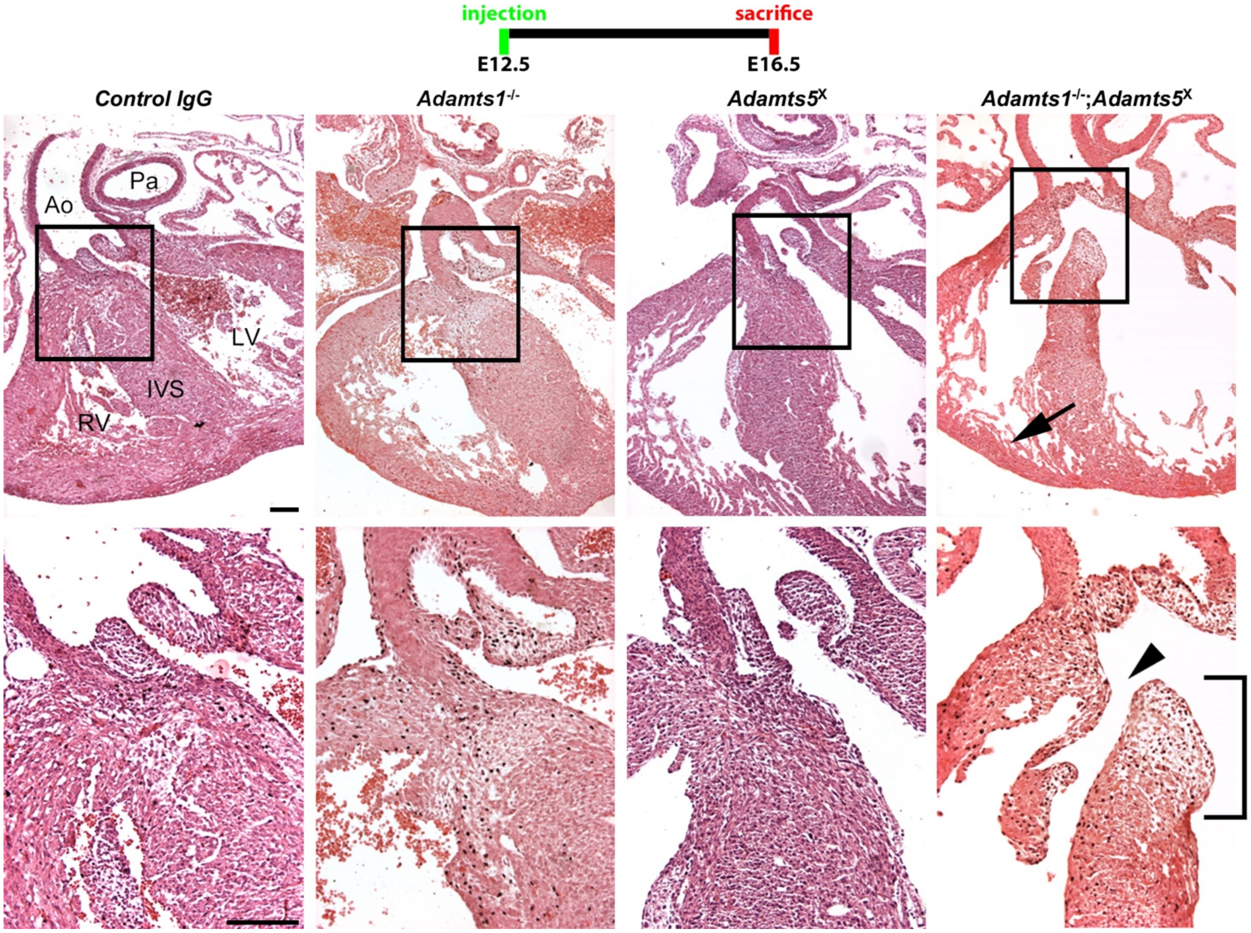

Figure S8. Combined pharmacological and genetic inactivation of ADAMTS1 and 5 results in rotational cardiac developmental defects and defective ventricular septal fusion. Hematoxylin and eosin-stained sections of E16.5 hearts of *Adamts1*$^{-/-}$ embryos that received either control or GSK12F4.1H7 antibody at E12.5 (insets show a representative image of the aortic valve). Combined inactivation resulted in an overriding aorta with associated VSD (arrowhead, far right panel; 100% penetrance – see Table 1) and a reduction in myocardial thickness (arrow) which was not observed in either *Adamts1*$^{-/-}$ or *Adamts5*^{X} hearts. The mesenchymal cap of the *Adamts1*$^{-/-}$;*Adamts5*^{X} ventricular septum is clearly visible (brackets). The

images are representative of N= 9 , 6, 7 and 8 for IgG control, *Adamts1*$^{-/-}$, *Adamts5*^{X} and *Adamts1*$^{-/-}$;*Adamts5*^{X} respectively. Scale bar = 100μm.

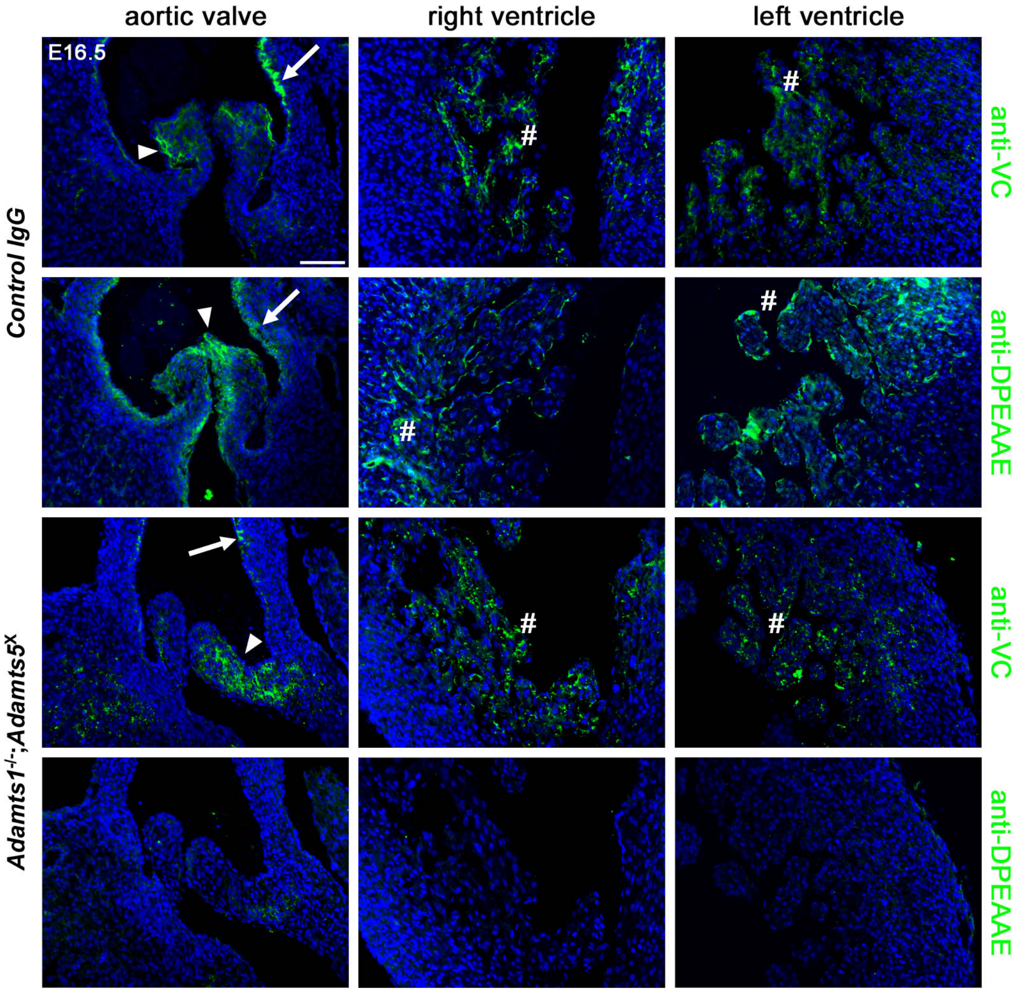

Figure S9. Versican cleavage is impaired by dual ADAMTS1 and ADAMTS5 inactivation. Versican (anti-VC) staining and versican cleavage detection (anti-DPEAAE) showed localization to outflow tract mesenchyme (arrow), valve leaflets (arrowhead), and trabecular myocardium (#) at E16.5. While VC staining is present in control IgG-recipient hearts, no DPEAAE staining was detected in *Adamts1*$^{-/-}$;*Adamts5*^{X} hearts. Images are representative of N= 4 for control IgG-treated and N=5 *Adamts1*$^{-/-}$;*Adamts5*^{X} hearts. Scale bar = 50μm.

A

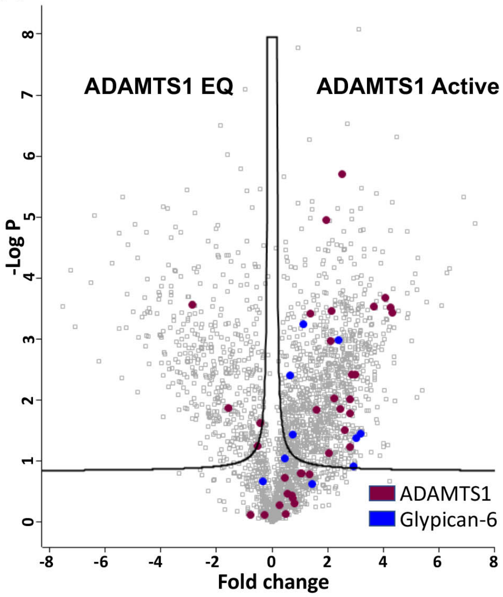

Glypican-6

MPSWIGAVILPLLGLLLSLPAGADVKARSCGEVRQAYGAKGFSLADIPYQEIAGEH
LRICPQEYTCCTTEMEDKLSQQSKLEFENLVEETSHFVRTTFVSR**HKKFDEFFRELL**
ENAEKSLNDMFVRTYGMLYMQNSEVFQDLFTELKRYYTGGNVNLEEMLNDFW
ARLLERMFQLINPQYHFSEDYLECVSKYTDQLKPFGDVPRKLKIQVTRAFIAAR**TF**
VQGLTVGREVANRV**SKVSPTPGCIR**ALMKMLYCPYCRGLPTVRPCNNYCLNVM
KGCLANQADLDTEWNLFIDAMLLVAERLEGPFNIESVMDPIDVKISEAIMNMQE
NSMQVSAKVFQGCGQPKPAPALRSARSAPENFNTRFRPYNPEERPTTAAGTSLD
RLVTDIKEKLKLSKKVWSALPYTICKDESVTAGTSNEEECWNGHSKARYLPEIMND
GLTNQINNPEVDVDITRPDTFIRQQIMALRVMTNKLKNAYNGNDVNFQDTSDE
SSGSGSGSGCMDDVCPTEFEFVTTEAPAVDPDRREVDSSAAQRGHSLLSWSLTCI
VLALQRLCR

B

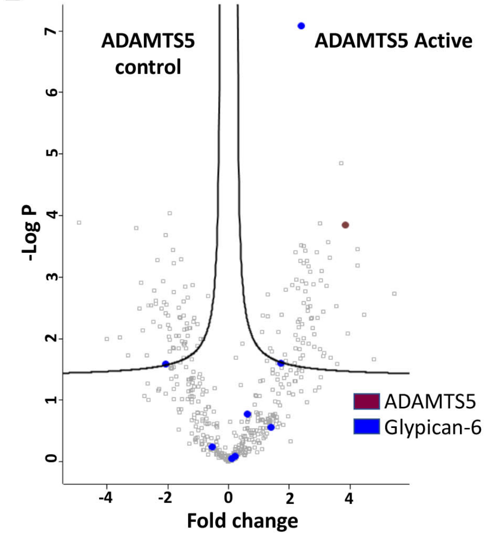

Glypican-6

MPSWIGAVILPLLGLLLSLPAGADVKARSCGEVRQAYGAKGFSLADIPYQEIAGEH
LRICPQEYTCCTTEMEDKLSQQSKLEFENLVEETSHFVRTTFVSRHKKFDEFFRELL
ENAEKSLNDMFVRTYGMLYMQNSEVFQDLFTELKRYYTGGNVNLEEMLNDFW
ARLLERMFQLINPQYHFSEDYLECVSKYTDQLKPFGDVPRKLKIQVTRAFIAARTF
VQGLTVGREVANRVSKVSPTPGCIRALMKMLYCPYCRGLPTVRPCNNYCLNVMK
GCLANQADLDTEWNLFIDAMLLVAERLEGPFNIESVMDPIDVKISEAIMNMQEN
SMQVSAKVFQGCGQPKPAPALRSARSAPENFNTR**FRPYNPEERPTTAAGTSLDR**
LVTDIKEKLKLSKKVWSALPYTICKDESVTAGTSNEEECWNGHSKARYLPEIMND
GLTNQINNPEVDVDITRPDTFIRQQIMALRVMTNKLKNAYNGNDVNFQDTSDE
SSGSGSGSGCMDDVCPTEFEFVTTEAPAVDPDRREVDSSAAQRGHSLLSWSLTCI
VLALQRLCR

Figure S10. Glypican-6 is cleaved in the presence of ADAMTS1 and ADAMTS5. A,B. Maroon dots identify putative autocatalytic ADAMTS1 and ADAMTS5 peptides whereas blue dots depict cleaved glypican-6 peptides arising in the presence of active ADAMTS1 or ADAMTS5. Dots above and outside the line are significant. Blue arrows in the glypican-6 amino acid sequences at right show the N-terminus of glypican-6 peptides (bold). Red lettering indicates the single cleaved peptide found by TAILS in wild type, but not *Adamts1*$^{-/-}$;*Adamts5*^{X} mouse heart.

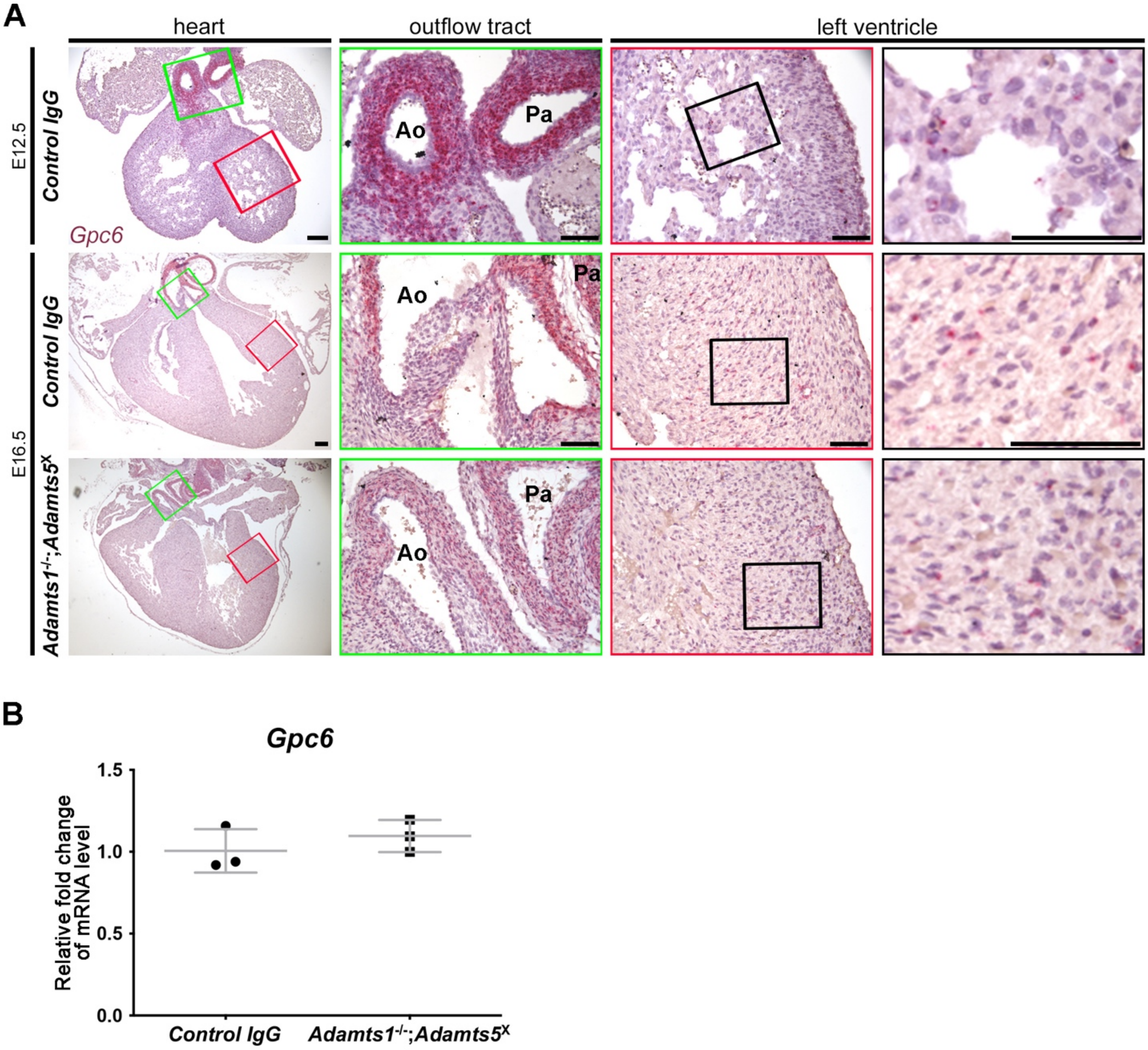

Figure S11. RNAscope but not RT-qPCR shows reduced *Gpc6* mRNA in *Adamts1*$^{-/-}$;*Adamts5*^{X} hearts. A. RNAscope in situ hybridization showed *Gpc6* expression (red) in E12.5 and E16.5 control cardiac outflow tract and ventricular myocardium. However, *Gpc6* signal was less intense in E16.5 *Adamts1*$^{-/-}$;*Adamts5*^{X} hearts. Images are representative of N = 3 from each group. **B.** RT-qPCR analysis of *Gpc6* mRNA in E16.5 wild type and *Adamts1*$^{-/-}$;*Adamts5*^{X} hearts shows comparable *Gpc6* expression. N=3. Error bars represent SEM. Student t-test.

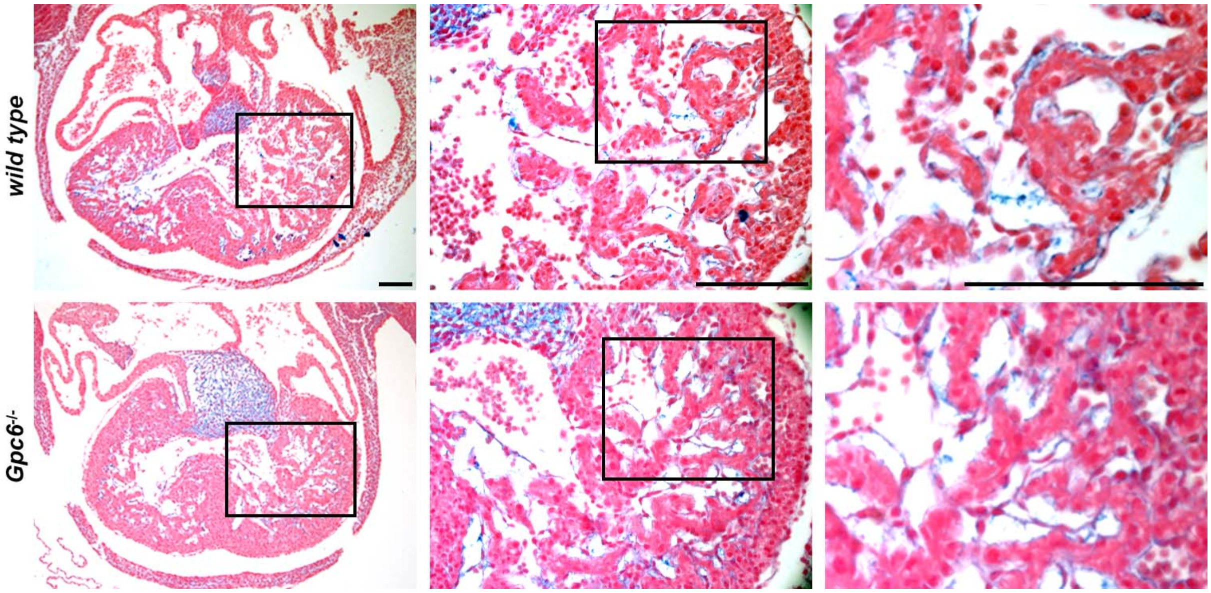

Figure S12. No change in proteoglycan content in *Gpc6*$^{-/-}$ hearts. Cardiac jelly in E12.5 *Gpc6*$^{-/-}$ hearts visualized by Alcian blue staining appears comparable to wild type littermates. Images are representative of N= 4. Scale bar = 100μm

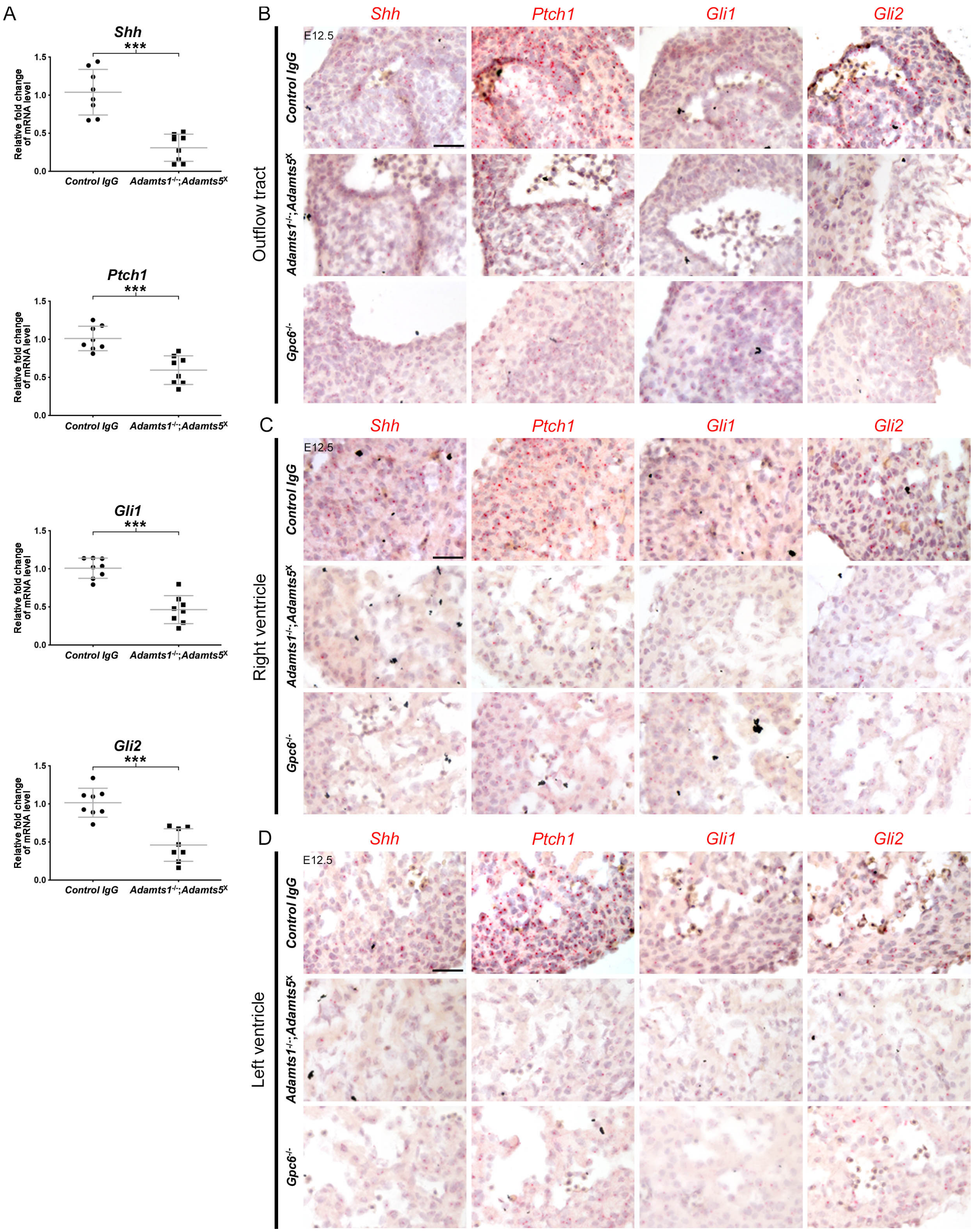
A
Shh
***
Ptch1
***
Gli1
***
Gli2
***
Relative fold change of mRNA level
1.5
1.0
0.5
0.0
Control IgG
Adamts1-/-;Adamts5X
B
C
D
Shh
Ptch1
Gli1
Gli2
E12.5
Control IgG
Adamts1-/-;Adamts5X
Gpc6-/-
Outflow tract
Right ventricle
Left ventricle

Figure S13. Developmental cardiac hedgehog signaling is suppressed by combined ADAMTS1 and ADAMTS5 inactivation and *Gpc6* knockout. A. RT-qPCR analysis shows reduction of Shh-responsive *Shh, Ptch1, Gli1 and Gli2* mRNAs in E16.5 *Adamts1*$^{-/-}$;*Adamts5*^{X} hearts compared to controls. Error bars represent SEM. ***p $\leq$ 0.001, Student t-test. n = 8 in each group. **B-D.** RNAscope in situ hybridization showed reduced signal (red) for *Shh*, *Ptch1*, *Gli1*, and *Gli2* mRNAs in E12.5 *Adamts1*$^{-/-}$;*Adamts5*^{X} and E12.5 *Gpc6*$^{-/-}$ hearts compared to control and wild type hearts. These RNAs were expressed in wild type cardiac outflow tract and heart valves as well as compact and trabecular myocardium. Images are representative of N=3 for each group. Scale bar = 25μm.

Table S1. Cardiac phenotypes in *Adamts1*$^{-/-}$;*Adamts5*^{X}, *Adamts1*$^{-/-}$ and *Adamts5*^{X} embryos at E16.5 following antibody injection at E12.5.

Genotype	VSD	OA	PS
Control IgG	0% (41)	0% (41)	0% (41)
Adamts1$^{-/-}$	0% (37)	0% (37)	0% (37)
Adamts5^{X}	0% (36)	0% (36)	100% (36)
Adamts1$^{-/-}$;*Adamts5*^{X}	86% (28)	86% (28)	100% (28)

Number in parenthesis denotes (n). VSD, ventricular septal defect; OA, overriding aorta; PS, pulmonic valve stenosis

Table S2. Statistically significant N-terminally labeled internal peptides from TAILS with higher abundance in control than in *Adamts1*$^{-/-}$;*Adamts5*^{X} hearts. Only peptides from molecules annotated by UniProt as secreted (S) or cell membrane (CM) proteins are shown.

Accession	Name	Modification	Preceding sequence . (= cleavage site) TAILS peptide	Control/ KO ratio	Adjusted p value	Cellular component
P05125	Atriopeptin II/natriuretic peptide A	1xDimethyl [N-Term]	GAAL(73).(74)SSLPEVPPWTGEVNPPLR	Identified only in control		S
P13020	Brevin/gelsolin	1xDimethyl [N-Term]	PSTM(50).(51)VVEHPEFLKAGKEPGLQIWR	Identified only in control		S
O88207	Collagen alpha-1(V) chain	1xDimethyl [N-Term]	PGMP(435).(436)ANQDTIFEGIGGPR	Identified only in control		S
O88569	Heterogeneous nuclear ribonucleoproteins A2/B1	1xDimethyl [N-Term]	FEEY(135).(136)GKIDTIEIITDR	Identified only in control		S
Q9R1V7	Metalloproteinase-like; disintegrin-like; and cysteine-rich protein 23/ADAM23	1xDimethyl [N-Term]	KRSS(397).(398)LSYFGGVCSRIR	Identified only in control		CM
P17742	Peptidyl-prolyl cis-trans isomerase A; N-terminally processed	1xDimethyl [N-Term]	KVKE(134).(135)GMNIVEAMER	2.595	0.001	S
			RVSF(22).(23)ELFADKVPKTAENFR	Identified only in control		S
			FELF(25).(26)ADKVPKTAENFR	Identified only in control		S
			FRAL(39).(40)STGEKGFGYKGSSFHR	Identified only in control		S
			HVVF(129).(130)GKVKEGMNIVEAMER	Identified only in control		S
			VKEG(135).(136)MNIVEAMER	Identified only in control		S
P34884	Phenylpyruvate tautomerase/Macrophage migration inhibitory factor	1xDimethyl [N-Term]	CALC(60).(61)SLHSIGKIGGAQNR	Identified only in control		S
			LCSL(62).(63)HSIGKIGGAQNR	Identified only in control		S
O54724	Caveolin-1	1xDimethyl [N-Term]	VLKA(340).(341)TEMVEVGPEDDEVGAER	Identified only in control		CM
Q9R087	Glypican-6	1xDimethyl [N-Term]	GLTN(442).(443)QINNPEVEVDITRPDTFIR	Identified only in control		CM

Q60651	T-cell surface glycoprotein Ly-49D	1xDimethyl [N-Term]	VIAL(50).(51)GILISLR	4.143	0.002	CM
Q9WTS6	Teneurin transmembrane protein 3	1xDimethyl [N-Term]	WRGA(780).(781)GCDVAMETLCTDSKDNEGDGLIDCMDPDCCLQSSCQNQPYCR	Identified only in control		CM
Q00623	Truncated apolipoprotein A-I	1xDimethyl [N-Term]	GQQL(65).(66)NLNLLENWDTLGSTVSQLQER	Identified only in control		S

Table S3. Peptides from proteins identified as putative ADAMTS1 substrates. N-terminally labeled internal peptides from the TAILS method with higher abundance in conditioned medium of ADAMTS1 overexpressing cells than ADAMTS1-EQ, expressing cells, each also expressing glypican-6 are shown. Only peptides from molecules annotated by UniProt as ECM or cell membrane (CM) with an extracellular domain are shown.

UniProt Accession	Protein name	Preceding sequence . (= cleavage site) TAILS peptide	Fold change ADAMTS1/EQ	Adjusted P-value	CC
Q9UHI8	A disintegrin and metalloproteinase with thrombospondin motifs 1	NVGR(108).(109)KSGSETPLPETDLAHCFYSGTVNGDPSSAAALSLCEGVR	2.856236	0.024248	ECM
		HCFY(126).(127)SGTVNGDPSSAAALSLCEGVR	2.232028	0.020279	
		FYSG(128).(129)TVNGDPSSAAALSLCEGVR	2.098745	0.029661	
		NGDP(134).(135)SSAAALSLCEGVR	2.122959	0.034552	
		AFYL(152).(153)LGEAYFIQPLPAASER	2.455164	0.01856	
		AAPG(175).(176)EKPPAPLQFHLLR	2.509463	0.05704	
		RRNR(191).(192)QGDVGGTCGVVDDEPRPTGK	1.605653	0.018371	
		PALQ(238).(239)GVGQPTGTGSIR	2.791351	0.017889	
		LFTR(359).(360)QDLCGSQTCDTLGMADVGTVCDPSR	2.801784	0.020132	
		DCSR(574).(575)TCGGGVQYTMR	4.071141	0.036818	
		YTMR(585).(586)ECDNPVPK	3.661226	0.035277	
		KTFR(623).(624)EEQCEAHNEFSKA	1.365665	0.034209	
		KTFR(623).(624)EEQCEAHNEFSKASF	2.80333	0.01231	
		KTFR(623).(624)EEQCEAHNEFS	4.243357	0.035217	
		VVLR(801).(802)YSGSSAALER	2.987858	0.024226	
		IEEW(864).(865)GECSKSCELGWQR	4.325845	0.034317	
		VECR(883).(884)DINGQPASECAKEV	2.033031	0.011254	
		YKKR(932).(933)SLKCLSH	2.598711	0.015051	
P02765	Alpha-2-HS-glycoprotein	LKLD(123).(124)GKFSVVYAKCDSSPDSAEDVR	2.911095	0.011654	ECM
		YAKC(133).(134)DSSPDSAEDVR	4.846763	0.047573	
		AKCD(134).(135)SSPDSAEDVR	2.983584	0.037021	
		KCDS(135).(136)SPDSAEDVR	3.810088	0.028623	
P01023	Alpha-2-macroglobulin	ALSK(1264).(1265)YGAATFTRTG	3.093129	0.032966	ECM
		ANGR(854).(855)QTVSWAVTPK	4.100193	0.036369	
P08758	Annexin A5	NAQR(51).(52)QEISAAFKTLFGR	1.377368	0.018172	M
P02649	Apolipoprotein E	AQAR(122).(123)LGADMEDVCGR	1.90754	0.025032	ECM
		VQYR(138).(139)GEVQAMLGQSTEELR	2.032308	0.020531	
		LQKR(177).(178)LAVYQAGAR	3.43369	0.034742	

Q8N139	ATP-binding cassette sub-family A member 6	SFCV(1311).(1312)QEGEILGLLGPNGAGKSSSIRMISGITKPTAGEVELK	3.513325	0.030033	CM
O94985	Calsyntenin-1	HFAR(666).(667)AASEFESSEGVFLFPELR	1.822803	0.022183	CM
Q9BQT9	Calsyntenin-3	CGFR(70).(71)LHGSGVPFEAVILDKATGEGLIR	2.501981	0.031546	CM
Q9BY67	Cell adhesion molecule 1	QTQR(236).(237)YLEVQYKPQVH	4.354948	0.038229	CM
Q00610	Clathrin heavy chain 1	LAQR(1499).(1500)LEKHELIEFR	2.304751	0.017413	CM
P01024	Complement C3	SVVR(842).(843)NEQVEIR	3.118867	0.080801	ECM
		QGTP(987).(988)VAQMTEDAVDAER	2.503157	0.022237	
P00751	Complement factor B	ISFH(131).(132)CYDGYTLR	4.122851	0.033188	ECM
P23142	Fibulin-1	EGTR(354).(355)CVDVDECAPPAEPCGKGHR	2.339958	0.051436	ECM
		KGHR(373).(374)CVNSPGSFR	3.260367	0.04786	
		KECR(63).(64)MVQEQCCHSQLEELHCATGISLANEQDR	1.973622	0.012193	
Q08380	Galectin-3-binding protein	DLSR(138).(139)ELSEALGQIFDSQR	3.202092	0.054752	CM
P06396	Gelsolin	GTSR(554).(555)EGGQTAPASTR	2.584364	0.022357	ECM
		SAKR(730).(731)YIETDPANR	3.742397	0.036184	
Q9Y625	Glypican-6	VSRH(103).(104)KKFDEFFR	2.921685	0.009179	CM
		EFFR(111).(112)ELLENAEKSLNDMFVR	3.179695	0.01444	
		IAAR(220).(221)TFVQGLTVGR	2.392686	0.029857	
		VANR(235).(236)VSKVSPTPGCIR	3.033738	0.013708	
P18065	Insulin-like growth factor-binding protein 2	PTIR(302).(303)GDPECHLFYNEQQE	1.674497	0.011468	ECM
P22692	Insulin-like growth factor-binding protein 4	ELVR(50).(51)EPGCGCCATCALG	2.741246	0.018878	ECM
P24593	Insulin-like growth factor-binding protein 5	LHGR(98).(99)GVCLNEKSYR	1.21078	0.034193	ECM
		KRIR(979).(980)TNLLQVCER	1.798381	0.012619	
P02751-10	Isoform 10 of Fibronectin	SWER(1198).(1199)STTPDITGYR	2.218061	0.041717	ECM
		TGYR(939).(940)VDVIPVNLPGEHGQR	1.913831	0.012672	
P08253-2	Isoform 2 of 72 kDa type IV collagenase	FPFR(221).(222)FQGTSYDSCTTEGR	2.154788	0.031384	ECM

O00468-2	Isoform 2 of Agrin	LVEK(1760).(1761)SAGDVDTLAFDGR	2.417507	0.025409	CM
P08697-2	Isoform 2 of Alpha-2-antiplasmin	LLSR(87).(88)LCQDLGPGAFR	5.127862	0.040687	ECM
Q9BY67-2	Isoform 2 of Cell adhesion molecule 1	IYFR(88).(89)DFRPLKDSR	2.948907	0.026823	CM
Q00610-2	Isoform 2 of Clathrin heavy chain 1	LVVR(838).(839)GQFSTDELVAEVEKR	3.785289	0.016193	CM
		CEKR(913).(914)DPHLACVAYER	3.389107	0.02902	
O43405-2	Isoform 2 of Cochlin	AVHR(81).(82)GVISNSGGPVR	1.60586	0.015605	ECM
P39060-1	Isoform 2 of Collagen alpha-1(XVIII) chain	ETWR(1476).(1477)TEAPSATGQASSLLGGR	1.937412	0.035232	ECM
Q12805-2	Isoform 2 of EGF-containing fibulin-like extracellular matrix protein 1	PENR(369).(370)CVCPVSNAMCR	1.432325	0.051299	ECM
Q13308-2	Isoform 2 of Inactive tyrosine-protein kinase 7	GKDR(591).(592)ILDPTKLGPR	1.474635	0.026341	CM
Q14112-2	Isoform 2 of Nidogen-2	NVDR(676).(677)VFALYNDEER	2.810945	0.031495	ECM
		GDGR(745).(746)NCVDENECATGFHR	2.281912	0.030999	
P55058-2	Isoform 2 of Phospholipid transfer protein	LPNR(200).(201)AVEPQLQEEER	2.391201	0.035807	ECM
Q99435-2	Isoform 2 of Protein kinase C-binding protein NELL2	CCPR(778).(779)CVTDPCQADTIR	1.833743	0.017465	ECM
Q9H4F8-2	Isoform 2 of SPARC-related modular calcium-binding protein 1	AKCR(76).(77)DPTLGVVHR	2.677325	0.035368	ECM
		TSTR(287).(288)YVMPSCESDAR	2.876138	0.051015	
P14209-2	Isoform II of CD99 antigen	NHPS(86).(87)SSGSFSDADLADGVSGGEGKGGSDGGGSHR	2.460532	0.037535	CM
		SGSF(91).(92)SDADLADGVSGGEGKGGSDGGGSHR	2.04643	0.025788	
P09496-2	Isoform Non-brain of Clathrin light chain A	QVDR(103).(104)LQSEPESIR	2.323242	0.019013	CM
		QMER(121).(122)LEALDANSR	1.903907	0.012324	
Q86UP2	Kinectin	LEQR(550).(551)LMQLMESEQKR	2.125882	0.028355	CM
P02788	Lactotransferrin	SNER(544).(545)YYGYTGAFR	6.885695	0.053309	ECM
Q01650	Large neutral amino acids	PKRR(10).(11)ALAAPAAEEKEEAR	1.368213	0.01622	CM

	transporter small subunit 1				
P14174	Macrophage migration inhibitory factor	SPDR(95).(96)VYINYYDMNAANVGWNNSTFA	1.347536	0.062656	ECM
Q9UMX5	Neudesin	FYGR(85).(86)GAPYNALTGKDSTR	1.332382	0.0152	ECM
Q15818	Neuronal pentraxin-1	KLGR(89).(90)CESQSTLDPGAGEAR	1.560563	0.013525	ECM
P14543	Nidogen-1	TIIR(1018).(1019)QDLGSPEGIAVDHLGR	1.858699	0.034321	ECM
		HLGR(1034).(1035)NIFWTDSNLDR	2.461763	0.025534	
		NLDR(1045).(1046)IEVAKLDGTQR	3.879961	0.01081	
		VNPR(1068).(1069)GIVTDSVR	2.768487	0.027665	
		GTNR(1133).(1134)AECLNPSQPSR	2.644848	0.013708	
		RTCR(1234).(1235)CPDNTLGVDCIEQK	5.193785	0.020383	
		VYYR(124).(125)EDLSPSITQR	3.562243	0.039853	
		SSTR(588).(589)EYTVTEPER	3.745614	0.053821	
		KILR(651).(652)YALSNSIGPVR	3.734152	0.026065	
		PGPR(691).(692)TQFTCECSIGFR	2.307914	0.009241	
P04746	Pancreatic alpha-amylase	WWER(77).(78)YQPVSYKLCTR	1.701218	0.022267	ECM
P26022	Pentraxin-related protein PTX3	ELGR(95).(96)LAESLARPCAPGAPAEAR	2.13	0.018677	ECM
		EAGR(148).(149)ALAAVLEELR	1.598173	0.025679	
Q15063	Periostin	REER(754).(755)IITGPEIKYTR	2.619159	0.013729	ECM
P10586	Receptor-type tyrosine-protein phosphatase F	IQSR(1007).(1008)TMPVEQVFAKNFR	1.874647	0.018632	CM
Q92743	Serine protease HTRA1	RAGR(30).(31)SAPLAAGCPDR	2.846988	0.049686	ECM
Q9H4F8	SPARC-related modular calcium-binding protein 1	DQER(232).(233)QSALEEAQQNPR	1.452155	0.01295	ECM
O76061	Stanniocalcin-2	PAIR(130).(131)EMVSQLQR	4.24712	0.047856	ECM
		KGER(263).(264)GSKSHPNAH	2.604708	0.027614	
P02786	Transferrin receptor protein 1	LIER(580).(581)IPELNKVAR	1.687448	0.014574	CM

Table S4: Peptides from proteins identified as putative ADAMTS5 substrates. N-terminally labeled internal peptides from the TAILS method with higher abundance in conditioned medium of ADAMTS5 overexpressing cells than empty vector-transfected cells, each also expressing glypican-6, are shown. Only peptides from molecules annotated by UniProt as ECM or cell membrane (CM) with an extracellular domain are shown.

UniProt Accession	Protein name	Preceding sequence .(= cleavage site) TAILS peptide	Fold Change ADAMTS5/vector	Adjusted p value	CC
Q9UNA0	A disintegrin and metalloproteinase with thrombospondin motifs 5	VQER(55).(56)AEPPGHPHPLAQR	Identified only in ADAMTS5 overexpressed cells		ECM
		VYTR(196).(197)EGFSFEALPPR			
		LYGR(286).(287)GLQHYLLTLASIANR			
P08758	Annexin A5	MVSR(277).(278)SEIDLFNIR	Identified only in ADAMTS5 overexpressed cells		CM
Q08380	Galectin-3-binding protein	DLSR(138).(139)ELSEALGQIFDSQR	Identified only in ADAMTS5 overexpressed cells		CM
Q9Y625	Glypican-6	FNTR(364).(365)FRPYNPEERPTTAAGTSLDR	Identified only in ADAMTS5 overexpressed cells		CM
P08195-2	Isoform 2 of 4F2 cell-surface antigen heavy chain	KSIR(203).(204)VILDLTPNYR	Identified only in ADAMTS5 overexpressed cells		CM
Q00610-2	Isoform 2 of Clathrin heavy chain 1	LQTR(584).(585)LLEMNLMHAPQVADAILGNQMFTHYDR	Identified only in ADAMTS5 overexpressed cells		CM
		NFGR(1227).(1228)LASTLVHLGEYQAAVDGAR			
Q13308-2	Isoform 2 of Inactive tyrosine-protein kinase 7	SFAR(227).(228)VVLAPQDVVVAR	Identified only in ADAMTS5 overexpressed cells		CM
P14543-2	Isoform 2 of Nidogen-1	IKQR(537).(538)FSGIDEHGHLTIDTELEGR	Identified only in ADAMTS5 overexpressed cells		ECM
		GTQR(923).(924)RVLFETDLVNPR	4.542748	0.043762	ECM
P10586-2	Isoform 2 of Receptor-type tyrosine-protein phosphatase F	SDTR(528).(529)IQLSWLLPPQER	Identified only in ADAMTS5 overexpressed cells		CM
Q03167-2	Isoform 2 of Transforming growth factor beta receptor type 3	PELR(375).(376)ILLDPGALPALQNPPIR	Identified only in ADAMTS5 overexpressed cells		CM
P10646-2	Isoform Beta of Tissue factor pathway inhibitor	IMKR(70).(71)FFFNIFTR	Identified only in ADAMTS5 overexpressed cells		ECM
P13473-2	Isoform LAMP-2B of Lysosome-associated membrane glycoprotein 2	LAIR(145).(146)IPLNDLFR	Identified only in ADAMTS5 overexpressed cells		CM
Q15758	Neutral amino acid transporter B(0)	DLAR(190).(191)NIFPSNLVSAAFR	Identified only in ADAMTS5 overexpressed cells		CM

Table S5. Cardiac phenotypes in E16.5 *Gpc6*$^{-/-}$ embryos.

Genotype	VSD	OA	PS	DORV
Wild type	0% (13)	0% (13)	0% (13)	0% (13)
Gpc6$^{-/-}$	63% (16)	56% (16)	55% (11)	25% (16)

Number in parenthesis denotes (n). VSD, ventricular septal defect; OA, overriding aorta; PS, pulmonic valve stenosis; DORV, double outlet right ventricle.

Table S6. Quantitative real-time PCR primers

Gene	Primer sequence
Adamts1	Forward: 5´-GAAGGCAAACGAGTCCGCTACA-3´
	Reverse: 5´-TTGGGTGTCCACTCTACAGTGG-3´
Adamts5	Forward: 5´-CTGCCTTCAAGGCAAATGTGTGG-3´
	Reverse: 5´-CAATGGCGGTAGGCAAACTGCA-3´
Gpc6	Forward: 5´-AGAGGTTGCCAACCGAGTTTCC-3´
	Reverse: 5´-TGCAAGGTCTCACAGTGGGCAA-3´
Shh	Forward: 5´-GGATGAGGAAAACACGGGAGCA-3´
	Reverse: 5´-TCATCCCAGCCCTCGGTCACT-3´
Ptch1	Forward: 5´-CCTCGCTTACAAACTCCTGGTG-3´
	Reverse: 5´-TGATGCCATCTGCGTCTACCAG-3´
Gli1	Forward: 5´-CTCAAACTGCCCAGCTTAACCC-3´
	Reverse: 5´-TGCGGCTGACTGTGTAAGCAGA-3´
Gli2	Forward: 5´-ACACTGTGGAGGACTGCCTACA-3´
	Reverse: 5´-GGCATCTCCATGCCACTGTCAT-3´
Gapdh	Forward: 5´-TGGAGAAACCTGCCAAGTATGA-3´
	Reverse: 5´-CTGTTGAAGTCGCAGGAGACA-3´

Table S7. Primary antibodies and HA-staining reagent

Antibody	Source/citation	Product number	Dilution
Anti-VC (versican)	PMID: 25122765	In-house	1:500
Anti-DPEAAE	ThermoFisher Scientific	PA1-1748A	1:400
Anti-versican GAGβ	Millipore	AB1033	1:500
HAbp	Millipore	385911	1:100
Anti-Gpc6	R&D	AF2845	1:100
Anti-Shh	DSHB	5E1-c	1:100
Anti-Gli1	Rockland	100-401-223	1:100
Anti-Ptch1	R&D	MAB41051	1:100
Anti-Acan	Millipore	AB1031	1:400
Anti-NITEGE	Invitrogen	PA1-1746	1:100
Anti-CLP	DHSB	9/30/8-A4-c	1:100
Anti-Fn1	Millipore	AB2033	1:500

Supplemental Videos

Supplemental Video 1. Synchrotron-imaged control E16.5 heart. The video pauses at the site of the fully formed ventricular septum.

Supplemental Video 2. Synchrotron-imaged *Adamts1*$^{-/-}$, *Adamts5*^{X} E16.5 heart. The video pauses at the site of the ventricular septal defect.
